# An Integrated Approach Combining Regulatory Tests in *tg*(*cyp19a1b:GFP*) Zebrafish Embryos to Assess Toxicity, Developmental Effects, and Estrogenic Activity of Chemicals: A Case Study with Bisphenol A Substitutes

**DOI:** 10.1021/acs.est.5c17875

**Published:** 2026-04-10

**Authors:** Florian Geffroy, Armelle Christophe, Benjamin Piccini, Nathalie Hinfray, Edith Chadili, Emmanuelle Maillot-Marechal, Xavier Cousin, Mélanie Blanc-Legendre, Thierry D. Charlier, Pascal Pandard, Selim Aït-Aïssa, François Brion

**Affiliations:** † 52805 Institut National de l’Environnement Industriel et des Risques (INERIS), Parc Technologique ALATA BP2, F-60550 Verneuil-en-Halatte, France; ‡ Université Paris Cité, Inserm, HealthFex, F-75006 Paris, France; § MARBEC, Université de Montpellier, CNRS, Ifremer, IRD, Inrae, F-34250 Palavas-Les-Flots, France; ∥ Université de Rennes, Inserm, EHESP, Irset (Institut de Recherche en Santé, Environnement et Travail), F-35000 Rennes, France; ⊥ ImPACcell Platform, Biosit, Université de Rennes, F-35000 Rennes, France

**Keywords:** *Danio rerio*, OECD test guidelines, FET, EASZY, new approach methodologies (NAMs), bisphenols, endocrine disruption, environmental hazard assessment

## Abstract

The use of efficient testing strategies for chemical hazard assessment is a current challenge in supporting regulatory requirements. Herein, we combined two zebrafish eleuthero-embryo assays, a refined Fish Embryo Acute Toxicity assay (FET, OECD TG 236) and the EASZY assay (OECD TG 250), both using transgenic tg­(*cyp19a1b:GFP*) embryos, to jointly assess acute toxicity, developmental effects, and estrogenic activity of bisphenol A (BPA) and ten substitutes. Several bisphenols were more toxic than BPA, inducing developmental effects on zebrafish embryos, with some showing potential teratogenicity. Increased GFP intensity was also detected for ten bisphenols, suggesting estrogenic activity. FET results guided the selection of sublethal concentrations for the EASZY assay, which confirmed estrogenic activity for all bisphenols except TCBPA, most being more estrogenic than BPA *in vivo* and requiring functional zfERs to induce brain aromatase. All bisphenols also activated zfERβ2 in a zebrafish-specific *in vitro* reporter gene assay, except BPS-MAE and BPS-MPE, which only induced brain aromatase *in vivo*. Overall, the combined FET and EASZY assays efficiently generated relevant data for hazard assessment of chemicals and provided further evidence that bisphenols modulate ER-dependent *cyp19a1b* expression during early zebrafish brain development, raising concerns about their potential short- and long-term adverse effects.

## Graphical abstract

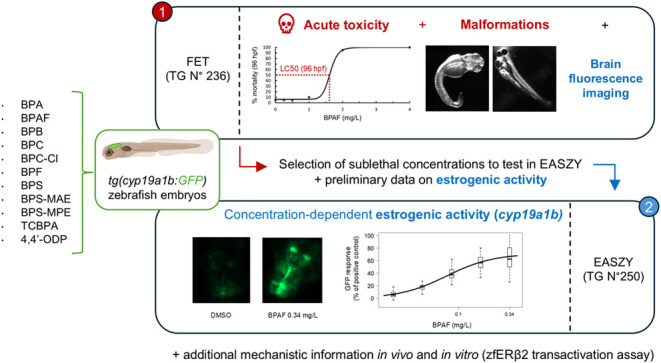

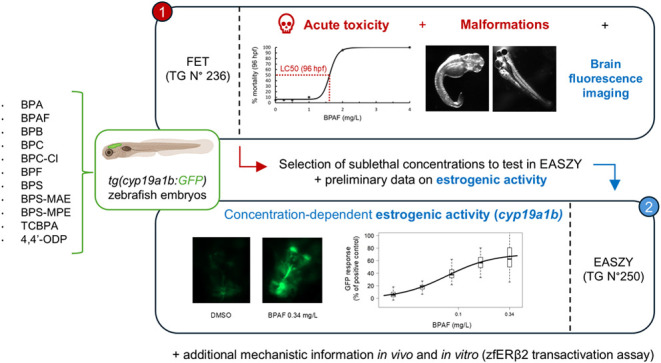

## Introduction

Bisphenol A (BPA) is a highly produced and well-recognized endocrine-disrupting chemical (EDC). It interacts with nuclear receptors in various cellular and animal models and consequently triggers adverse health effects in humans and wildlife.^1,2^ As a result, the use and production of BPA have been restricted or banned in several countries. For example, the European Food Safety Authority (EFSA) lowered its tolerable daily intake by a factor of 20 000 in 2023 (from 4 μg/kg body weight/day to 0.2 ng/kg body weight/day)^3^ and the EU banned its use in food-contact materials in 2024.^4^ Other bisphenols, structurally or functionally related to BPA, intended to replace it for various industrial applications, are increasingly used and detected in humans and in the environment.^5–8^ Some of these substitutes, such as BPF, BPS, or BPB, have already been investigated, highlighting their endocrine activity via several nuclear receptors and related signaling pathways.^9–11^ These molecules have been shown to affect reproductive, metabolic, and developmental parameters in various models, thereby raising concerns about their health effects in humans and wildlife and stimulating the regulatory needs to further evaluate their endocrine-disrupting properties.^12^

However, information on the potential endocrine activity of numerous bisphenols is only partially available, preventing assessment of the risks they could potentially pose to humans and wildlife. From this perspective, the implementation of new approach methodologies (NAMs) and integrated testing strategies for the health and environmental hazard assessment of chemicals is still needed.

During the past decade, the zebrafish embryo has become an essential alternative model to animal testing in (eco)­toxicology studies. At the regulatory level, two zebrafish eleuthero–embryo assays have been adopted as OECD test guidelines (TG), one to assess the acute toxicity of chemicals, i.e., the Fish Embryo Acute Toxicity test (FET) (TG N°236),^13^ and another to assess the estrogenic activity of test chemicals at nontoxic concentrations, i.e., the EASZY assay (TG N°250).^14^ EASZY is the first eleuthero–embryo screening assay designed to inform on the estrogenic activity of chemicals acting through estrogen receptors (ERs) in transgenic (tg) *tg*(*cyp19a1b:GFP*) zebrafish. It is also the only OECD test guideline allowing us to assess the endocrine activity of test chemicals within the developing brain in a vertebrate model.

Herein, we aimed at assessing the toxicity, developmental effect, and estrogenic activity of BPA and ten substitutes, combining both a refined FET assay and the EASZY assay using *tg*(*cyp19a1b:GFP*) zebrafish embryos. This testing strategy was complemented using a zebrafish-specific *in vitro* reporter gene assay^15^ to gain additional mechanistic information on the ability of bisphenols to bind and activate zfERβ2, one of the first of the three ER subtypes to emerge in the brain during zebrafish embryonic development.^16^ The data collected are discussed with respect to existing data and potential regulatory outcomes.

## Materials and Methods

### Zebrafish Husbandry

Sexually mature transgenic *tg*(*cyp19a1b:GFP*) zebrafish (*Danio rerio)* ^17^ were used as breeding stocks. The fish were raised in a recirculating water system (Techniplast, Décines-Charpieu, France) at 27 °C under a controlled photoperiod (14 h light/10 h dark cycle). They were fed twice a day with dry food (Special Diet Services, Augy, France) and freshly hatched *Artemia salina* nauplii (Ocean Nutrition, Essen, Belgium). For breeding, a spawning tray was placed in each aquarium with a sex ratio of 2:1 (male:female). Spawning was stimulated by light the following morning, and eggs were collected and cleaned. Zebrafish reproduction was conducted in accordance with Directive 2010/63/EU, under experimental platform agreement number F6076902.

### Chemicals and Reagents

Bisphenols were purchased from Sigma-Aldrich (Saint-Quentin Fallavier, France), except TCBPA (ABCR, Karlsruhe, Germany), BPS-MPE (Santa Cruz Biotechnology, Dallas, TX), 4,4’-ODP (TCI Europe n.v., Paris, France), and BPS-MAE (Ark Pharm. Inc., Libertyville, IL). 17β-Estradiol (E2) was purchased from Sigma-Aldrich (Saint-Quentin Fallavier, France), and ICI 182,780 (ICI) was purchased from Tocris Bioscience (Bristol, UK). All relevant information about the selected chemicals is summarized in Table 1. Chemicals were dissolved in dimethyl sulfoxide (DMSO, purity 99.5%, Sigma-Aldrich). Reconstituted water^18^ was prepared every week for controls and preparation of testing chemicals.

**Table 1:**
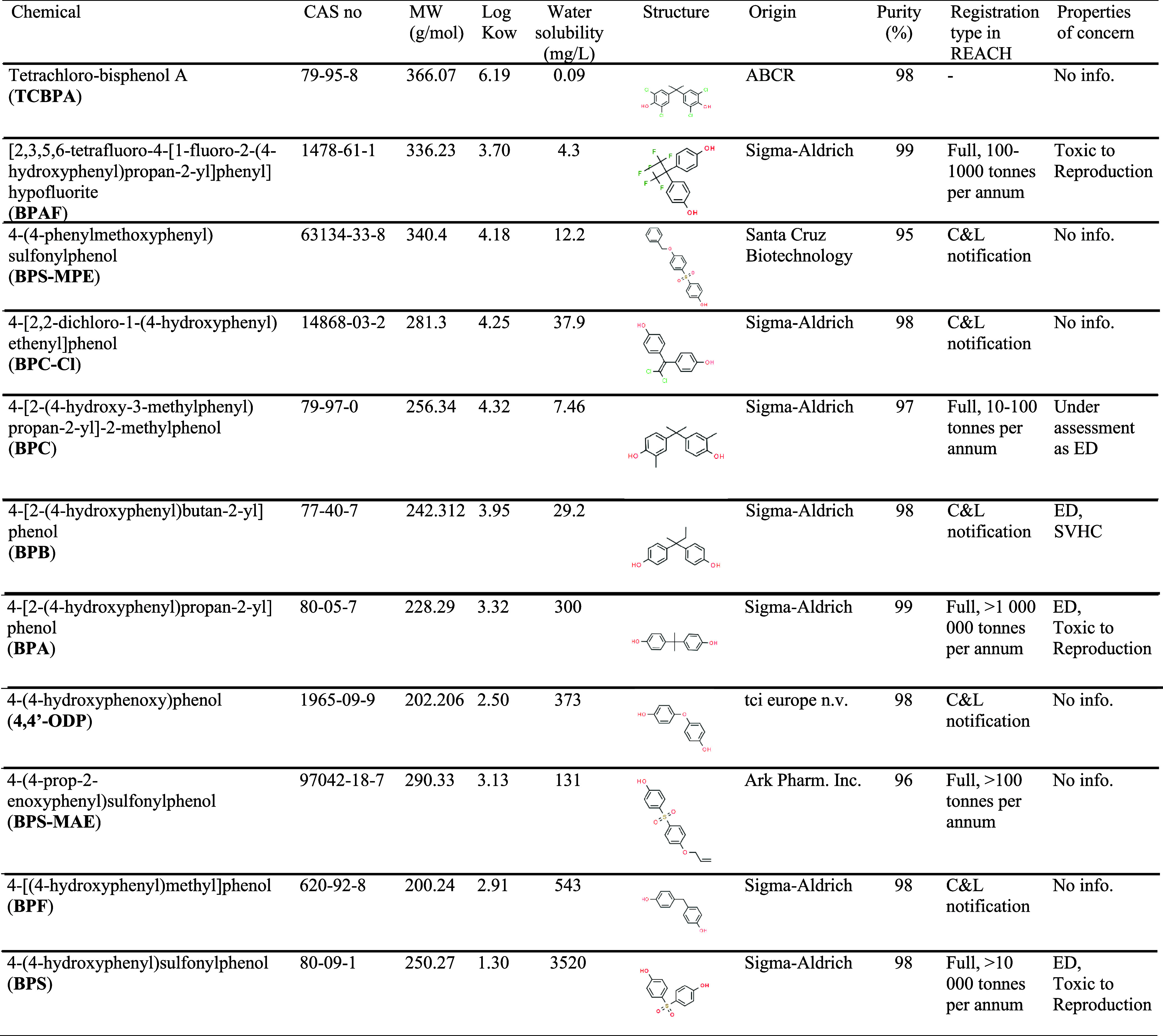
List of Bisphenols Tested and General Information^a^

a Log *K*_ow_ data were obtained from Adamovsky et al.,^8^ except for BPS-MAE, 4,4’-ODP, and BPC-Cl, which were obtained from ChemSpider. Water solubility data were compiled from Epi-Suite (US-EPA): experimental values when available (i.e., for BPA), and predictions from the WSKOWWIN model if not.

### Refined Fish Embryo Toxicity Tests Using *tg*(*cyp19a1b:GFP*) Zebrafish Embryos

All the tests were conducted according to OECD TG 236,^13^ except for the temperature (27 ± 2 °C), which was aligned with the OECD TG 250 ^14^ (see below). Exposures were carried out in presaturated 24-well plates containing one embryo per well in 2 mL of test solution. The exposure was performed under semistatic conditions, with daily renewal of the test solution. The concentration ranges tested, along with a control with the same percentage of solvent alone, are recapitulated in Table S1. All exposure concentrations are reported as nominal. During the time course of the refined FET tests, embryos were observed after 8 h (coagulation), 24 h (lack of somite formation and nondetachment of the tail), and 48, 72, and 96 h of exposure (nondetection of the heartbeat). Based on these criteria, the cumulative mortality rate was calculated at the end of the 96 h of exposure, and lethal concentrations inducing 50% mortality (LC_50_) were derived. Moreover, hatching rate and nonlethal developmental effects (i.e., yolk deformity, growth retardation, spontaneous movement, edema, blood tail circulation, heart rate, pigmentation, head and tail/spinal malformations, and hemorrhage) were monitored daily. Tail/spinal malformations included lordosis, kyphosis, scoliosis, and reduced tail length. At the end of exposure (96 h), living transgenic zebrafish embryos from test concentrations that do not induce more than 10% of mortality were collected for fluorescence imaging in order to acquire preliminary information about the potential estrogenic activity of the test chemicals. To do so, each embryo was photographed and analyzed as described by Brion et al.^19^ At the end of the refined FET assay, all living zebrafish embryos were euthanized using a protocol combining anesthesia induced by 168 mg/L tricaine (Pharmaq, delivered from Vetofish, Chateauneuf-les-Martigues, France) and the addition of bleach solution (sodium hypochlorite 6.15%; 1 volume of bleach to 5 volumes of water) to induce death.^14^

### EASZY Assay

The tests were conducted according to OECD TG 250.^14^ Newly fertilized *cyp19a1b*-GFP eggs were transferred to glass crystallizing dishes and exposed for 96 h in a humidified climate chamber (27 ± 2 °C) under semistatic conditions with a total renewal of the test solutions each day. Each condition consisted of three crystallizing dishes, each containing 7 embryos and 15 mL of test medium. Embryos were exposed to a range of nonlethal concentrations of chemicals as determined previously based on the refined FET assays. The tested concentration ranges of bisphenols, along with a control with the same percentage of solvent alone, are summarized in Table S2. All exposure concentrations are reported as nominal. At the end of exposure, transgenic zebrafish embryos were collected for fluorescence imaging to quantify GFP intensity. At the end of the experiments, all living zebrafish embryos were euthanized as described above. For coexposure experiments, embryos were exposed to bisphenols in the presence of 1 μM of the ER antagonist ICI 182,780 (ICI). ICI was added together with bisphenols from 0 to 4 dpf (days post fertilization). For BPC-Cl, BPS-MAE, and BPS, an additional coexposure experiment was performed by first exposing zebrafish embryos to 1 μM ICI alone from 0 to 2 dpf, followed by coexposure with bisphenols and ICI from 2 to 4 dpf. In all coexposure experiments, the final DMSO concentration was 0.02% (v/v).

### *In Vitro* Assay

The ZELH-zfERs reporter cell line was previously established after a two-step stable transfection of the zebrafish hepatic cell line ZFL with the luciferase gene under the control of ERE (yielding the ZELH cell line that expresses no functional ER) and the zebrafish ER subtype β2 (yielding the ZELH-zfERβ2 cell line).^15^ Cells were cultured, exposed to chemicals for 72 h, and measured for luciferase activity exactly as previously described.^9,15^ For each chemical, serial dilutions were prepared in DMSO and tested in three independent experiments. The final DMSO concentration in the culture medium was always 0.1% v/v. All tested concentrations refer to nominal concentrations.

### Data Analysis

Concentration–mortality curves were modeled using the Regtox Microsoft Excel macro, which applies the Hill model (http://www.normalesup.org/~vindimian/fr_index.html) to derive lethal concentrations inducing 10 and 50% of mortality (LC_10_ and LC_50_ values) after 96 h of exposure, with 95% confidence intervals (95% CI). For each treatment, the lowest-observed effect concentration (LOEC) was determined for each malformation criterion if at least 10% of individuals were affected and if the parameter exhibited a concentration- or time-dependent response. Based on these observations, the percentage of embryos exhibiting the most prevalent malformation at the end of the 96 h exposure for each concentration was used to model concentration–response curves and derive the effective concentration leading to 50% of malformed embryos (EC_50_). This analysis considered all nonlethal developmental end points monitored in the refined FET assay (see above), except effects on hatching, pigmentation, spontaneous movement, and blood circulation. A teratogenic index (TI) was then calculated as the ratio LC_50_/EC_50_.

For fluorescence imaging, GFP fold induction was expressed as mean ± SEM, and values were compared to the DMSO control using one-way ANOVA followed by Dunnett’s posthoc test. For the concentration–response analysis, fold induction values were normalized to the internal positive control of each experiment (0.05 nM EE2) and expressed as a percentage of the EE2 response. Normalized data were modeled using the *drc* package (v3.0.1)^20^ in R (v4.3.3),^21^ with a three-parameter log–logistic model (LL2.3) to derive relative EC_10_, EC_20_, and EC_50_ values (% of the modeled upper asymptote) and absolute EC_10_ (% of the positive control response: 0.05 nM EE2, also referred to as PC_10_). When concentration–response curves did not reach a clear plateau (BPB, BPC, BPF, 4,4’-ODP, and BPS-MPE), the upper asymptote parameter was fixed to the maximal observed effect of the tested substance.

For cell assays, results were expressed as % relative to the maximal luciferase activity induced by the positive control E2 (10 nM), and concentration–response curves were modeled using the RegTox Excel macro to derive relative EC_50_ values. In the case of incomplete concentration–response curves (i.e., no plateau observed), the maximal effect parameter was fixed to 100% activation and an absolute EC_50_ value, also referred to as PC_50_ (concentration inducing 50% of the maximal positive control response), was determined.

### Prediction of FET LC_50_ Baseline Toxicity and Assessment of Toxic and Sensitivity Ratios

Baseline toxicity LC_50_ was calculated using a zebrafish embryo QSAR (Quantitative Structure–Activity Relationship) from Klüver et al.,^22^ based on the log *K*_ow_ (logP, partition coefficient), with the following eq (eq 1), which we adapted in eq 2 to directly obtain LC_50_ in mg/L based on the molecular weight (MW) of the different bisphenols (Table 1)­

1 $$\mathrm{FET}\;\log1/{\mathrm{LC}}_{50}\left(\mathrm{mM}\right)=0.99\times \log\;K_{\mathrm{ow}}-2.02$$

2 $$\mathrm{FET}\;{\mathrm{LC}}_{50}\left(\mathrm{mg/L}\right)=\mathrm{MW}\times 1/10^{\left(0.99\times \log K_{\mathrm{ow}}-2.02\right)}$$

Baseline toxicity and experimental FET LC_50_ values were used to calculate toxic ratios (TR = predicted baseline toxicity LC_50_/experimental FET LC_50_). Compounds with TR < 10 were considered as baseline toxicants.^22^

Acute toxicity LC_10_ (FET assay) and estrogenicity absolute EC_10_ (EASZY assay) were used to calculate sensitivity ratios (SR = acute toxicity LC_10_/estrogenicity absolute EC_10_). The use of absolute EC_10_ ensures comparability across substances, including those that did not reach 50% of the EE2 positive control response.

All of the data generated in this study are compiled in the Supporting Information (Data set S1).

### Hierarchical Clustering

Malformation hierarchical clustering of bisphenols was performed based on their phenotypic fingerprints using Euclidean distance and Ward’s method. For each substance, the percentage of embryos affected per end point was calculated at the time point showing the maximal effect. Only concentrations ranging between the first concentration with >10% effect (for any end points) and the highest nonlethal concentration were considered to avoid nonspecific phenotypic effects. Heatmap and clustering were done in R (v4.3.3)^21^ using the *pheatmap* (v4.3.3)^23^ and *stats* (v1.0.12) packages.

## Results and Discussion

### Most Bisphenols Are More Toxic than BPA in the Fish Embryo Toxicity Test

In *tg*(*cyp19a1b:GFP*) zebrafish embryos, the LC_50_(96h) value for bisphenol A was found to be 14.14 mg/L. For the other bisphenols tested, variable acute toxicity was observed in the FET tests, with nominal LC_50_(96h) ranging from 0.88 mg/L for TCBPA to 35 mg/L for BPF, while BPS was not toxic at the highest tested concentration (400 mg/L) (Table 2). These results allow the classification of bisphenols from the most toxic to the less toxic according to their 96h LC_50_: TCBPA > BPAF > BPS-MPE > BPC-Cl > BPC > BPB > BPA > 4,4’-ODP > BPS-MAE > BPF > BPS. For the seven out of 11 bisphenols for which previous data were available, our results are in agreement with LC_50_ values reported in the literature using wild-type zebrafish embryos (Table 2), thereby supporting the suitability of *tg*(*cyp19a1b:GFP*) embryos to provide reliable acute toxicity data in the refined FET test. This is further supported by the LC_50_ obtained for the FET positive control chemical (3,4-DCA) in *tg*(*cyp19a1b:GFP*), which is very close to the mean LC_50_ value reported in the OECD TG 236 validation report,^24^ respectively, 2.46 and 2.7 mg/L. For the four chemicals for which no information was previously available, namely BPS-MPE, BPC-Cl, 4,4’-ODP, and BPS-MAE, this study provides LC_50_ in the 3.17–20.07 mg/L range and reveals that two of them (i.e., BPS-MPE and BPC-Cl) are more toxic than BPA. Four out of 11 bisphenols have log *K*_ow_ >4 and can be considered as difficult-to-test substances. We addressed whether water solubility could influence the acute toxicity values by comparing the experimental LC_50_ values to their maximum water solubility. Except for BPC and TCBPA, bisphenol LC_50_ values were below half of their maximum water solubility. This indicates the reliability of the acute toxic effect concentrations for nine bisphenols and suggests an underestimation of the acute toxicity for BPC and TCBPA, whose LC_50_ values should be interpreted with caution.

**Table 2:** Comparative Acute Toxicity of Bisphenols in Zebrafish Embryos: Experimental FET LC_50_ (Nominal Concentrations), Literature, QSAR Predictions, and Toxic Ratios^a^

	LC_50_ (96h) [mg/L, nominal]				toxic ratio
chemicals	experimental (FET test)	literature	references	predicted (QSAR)	(LC_50_ predict./LC_50_ exp.)
TCBPA	0.88 [0.85;0.89]	≈ 1	Song et al.^1,25^	0.028	0.032
BPAF	1.63 [1.24;1.66]	2.77	Arrokhman et al.^26^	7.65	4.69
		2.04	Ren et al.^27^		
		1.95 [1.72–2.23]	Mu et al.^28^		
		1.6 (0.09)	Moreman et al.^29^		
		1.16	Gao et al.^30^		
BPS-MPE	3.17 [2.95;3.43]	no data		2.59	0.82
BPC-Cl	4.18 [3.85;6.07]	no data		1.83	0.44
BPC	4.92 [2.84;8.53]	2.22	Gao et al.^30^	1.42	0.28
BPB	6.85 [6.28;6.89]	6.79	Arrokhman et al.^26^	3.12	0.45
		3.88	Ren et al.^27^		
		3.52	Gao et al.^30^		
BPA	14.14 [10;20]	13	Arrokhman et al.^26^	12.35	0.89
		12 (0.22)	Moreman et al.^29^		
		11.69	Gao et al.^30^		
		10.4 [9.44–11.58]	Mu et al.^28^		
		9.82	Blanc et al.^31^		
		8.041 [7.846;8.24]	Chow et al.^32^		
		5.82	Ren et al.^27^		
4,4’-ODP	17.05 [10.82;17.21]	no data		70.9	4.16
BPS-MAE	20.07 [14.77;20.49]	no data		24.22	1.21
BPF	35.07 [33.99;35.07]	32 (0.55)	Moreman et al.^29^	27.58	0.79
		24.5	Gao et al.^30^		
		24	Arrokhman et al.^26^		
		19.6 [18.47–20.67]	Mu et al.^28^		
		7.40	Gu et al.^33^		
		7.40	Ren et al.^27^		
BPS	>400	>100	Moreman et al.^29^	1353.35	NA
		>50	Mu et al.^28^		
		>100	Blanc et al.^31^		
		323 [308–339]	Gu et al.^34^		
		323	Ren et al.^27^		

a LC_50_ (96 h) values (in nominal concentrations) of the 11 tested bisphenols were determined experimentally using the refined FET assay and compared with values predicted by a QSAR baseline toxicity model. Toxic ratios (TR = LC_50_ predicted/LC_50_ experimental) are reported. Available LC_50_ (96 h) collected from the literature using the FET test are reported when available. Values in brackets indicate 95% confidence intervals; values in parentheses indicate SEM. See Figure S1 for complete modeled curves.

To further explore whether the acute toxicity of bisphenols could be explained by baseline toxicity (i.e., a nonspecific narcotic effect driven by hydrophobicity and cell membrane accumulation of chemicals), LC_50_ values were also predicted using a QSAR model from Klüver et al.,^22^ based on the hydrophobicity (log *K*_ow_) of chemicals. Although recent studies use log *K*_lipw_ and log *D*_lipw_ to broaden QSAR applicability domain,^22,35^ we used log *K*_ow_ as a first-tier descriptor, consistent with classical models for nonpolar and polar narcosis^36^ and due to its broader availability in the literature for bisphenols. The baseline toxicity QSAR is formally applicable to chemicals with log Klipw <4, which may result in unreliable predictions for the most hydrophobic bisphenols. We therefore distinguished bisphenols with a log *K*_ow_ <4 from those with a log *K*_ow_ >4 (Figure 1). By comparing observed and predicted LC50 and determining the Toxic Ratio (TR: LC50 predicted/LC50 experimental) (Table 2), we found that 4 out of 10 compounds were within a 2-fold range of deviation and 7 out of 10 within a 4-fold range of deviation (Figure 1), supporting a hydrophobicity-driven toxicity potential of bisphenols. An exception was noticed for TCBPA, which had a 32-fold higher predicted baseline toxicity than the experimental value (TR = 0.032). As baseline toxicity represents the minimum expected toxicity for organic chemicals, this discrepancy confirms the unreliability of the baseline toxicity QSAR model for highly hydrophobic compounds such as TCBPA (log *K*_ow_ = 6.19). Nevertheless, all BPA substitutes showed a TR < 10 (Table 2, Figure 1), suggesting they primarily act as baseline toxicants. These findings are consistent with the literature showing that endocrine active substances often appear as baseline toxicants in acute toxicity tests.^35,37^ However, this does not necessarily reflect their chronic toxicity, as EDCs can interfere with growth, reproduction, or development at concentrations well below those causing acute lethality in embryos.^38^ This reinforces the need to investigate more specific end points for such substances, including developmental malformations and estrogenic activity, for example, through investigation of ER-regulated brain aromatase *cyp19a1b* modulation.

**Figure 1:**
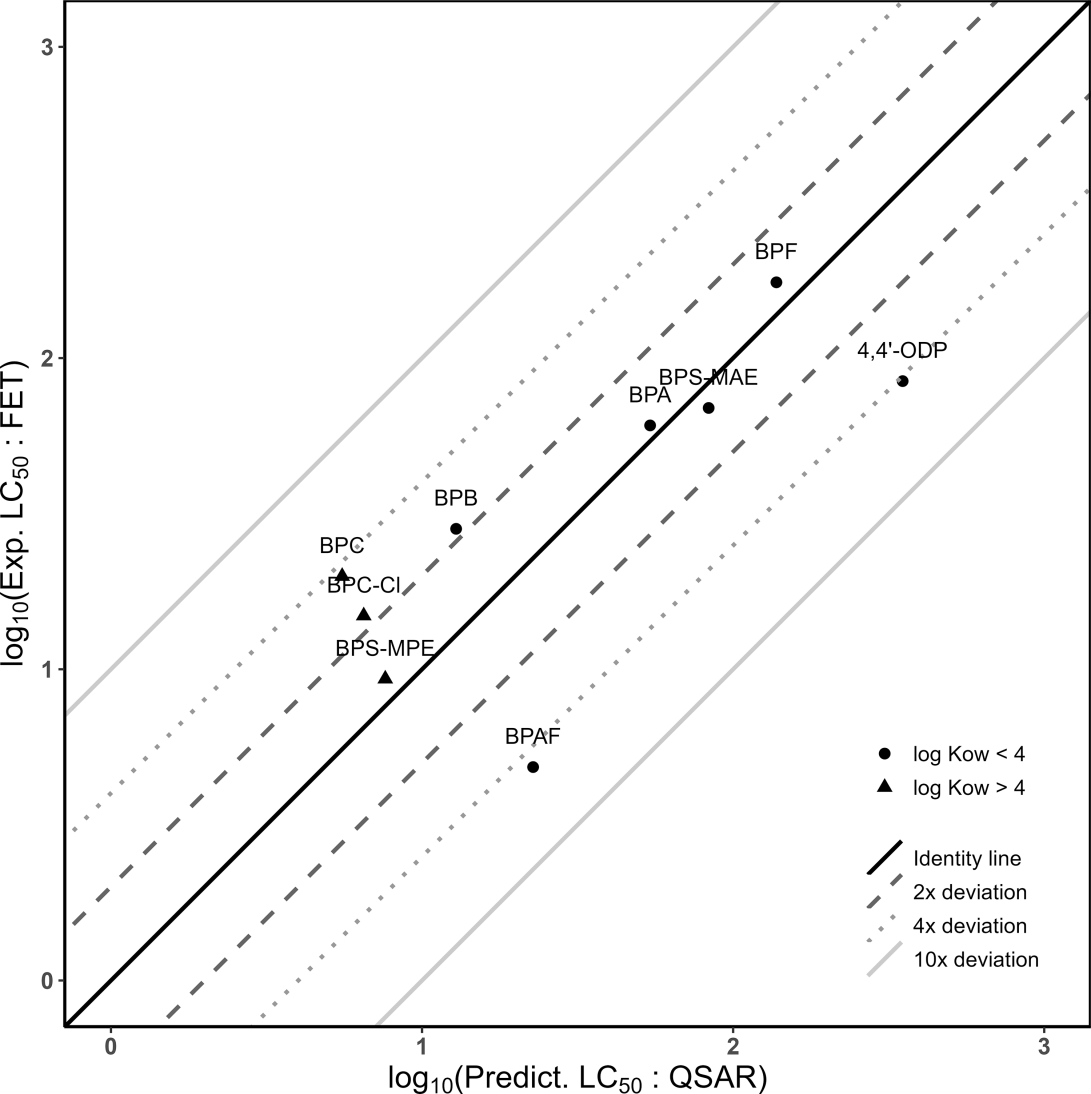
Comparison between experimental LC_50_ (nominal concentrations) and baseline toxicity QSAR-predicted LC_50_ for bisphenols in the Fish Embryo Toxicity (FET) tests. Experimental LC_50_ values were obtained following OECD TG 236 (FET), and predicted LC_50_ values were derived from a fish baseline toxicity QSAR.^22^ Each point represents a bisphenol. Circles indicate compounds with log *K*_ow_ <4 (within the baseline toxicity QSAR applicability domain), whereas triangles indicate compounds with log *K*_ow_ >4, for which predictions should be interpreted with caution due to increased uncertainty. The identity line (1:1) and deviation lines (2-, 4-, and 10-fold) illustrate prediction accuracy. TCBPA (TR = 0.032, less toxic than predicted) was not shown in the plot to improve readability. LC_50_ values (μM) are shown on a log10 scale to better visualize fold differences.

### Bisphenols Induce Developmental Effects on Zebrafish Embryos

Morphological and physiological changes were also recorded to assess more precisely the potential developmental toxicity of bisphenols. Most of them caused a reduced hatching success, frequently accompanied by a delayed hatching time (Figure S2). Interestingly, an exception was noted for BPS-MPE and BPS-MAE, for which an advance in hatching time was observed (Figure S2). It should be noted that lower concentrations of several bisphenols, including BPA, BPS, and BPAF have previously been shown to accelerate hatching rate in zebrafish.^39,40^ This impact of bisphenols on hatching deserves further investigation, as hatching is a finely regulated physiological process, involving both neuroendocrine circuits^41^ and the hatching gland.^42^ Furthermore, all the bisphenols studied induced other developmental effects of varying nature depending on the test chemicals, developmental stages, and exposure nominal concentrations. The most frequently observed phenotypic responses in bisphenols-exposed zebrafish were deformity of the yolk, edema, heart rate disruption, tail/spinal malformation, and hemorrhage (Table 3), consistent with effects previously reported after bisphenol exposure (Table S3). Several bisphenols elicited similar phenotypic response spectra, suggesting similar modes of action on developmental processes, with the notable exception of BPS, which did not show developmental effects except for yolk deformity observed at the highest concentration tested (Table 3). Among the recorded responses, reduction of pigmentation emerged as a specific and recurrent effect, reported for 7 out of the 11 bisphenols tested, i.e., BPA, BPF, BPS-MAE, BPS-MPE, BPC-Cl, TCBPA, and 4,4’-ODP (Figure S3). Altered pigmentation was previously reported in zebrafish embryos exposed to BPA and BPF, with a more pronounced effect following BPF exposures, in agreement with our results.^29,43^ In the present study, bisphenols-induced depigmentation occurred at varying concentrations and intensities depending on the bisphenols tested (Table 3, Figure S3), with more specific effects (i.e., at concentrations lower than acute toxicity) for BPA, BPF, and 4,4’-ODP. Strikingly, a complete depigmentation was observed in 4,4’-ODP-exposed embryos from a concentration as low as 0.99 mg/L. This effect likely reflects their ability to disrupt the melanin biosynthetic pathway,^43^ with a high efficiency for 4,4’-ODP as compared to others.

**Table 3:** Developmental Toxicity of Bisphenols in Zebrafish Embryos: LOECs, Malformation EC_50_, and Teratogenic Indexes (TI)^a^

	LOECs (mg/L, nominal)										
end points	TCBPA	BPAF	BPS-MPE	BPC-Cl	BPC	BPB	BPA	4,4′-ODP	BPS-MAE	BPF	BPS
deformity of yolk	1	1	5.56	0.99	2.84	10	20	2.96	11.1	15	400
growth retardation							20	8.89			
no spontaneous movement^b^	2	2		2.96		10	10		33.3	60	
edema	0.5		5.56	0.99	2.84	5	10	0.99	33.3	15	
no blood tail circulation^b^	1			8.89	8.53			8.89	33.3	60	
heart rate disruption	1	2	5.56	8.89	8.53	10	20	2.96	33.3	60	
no/Less pigmentation^b^	1		5.56	8.89			10	0.99	33.3	3.75	
head malformations	1	2				10		2.96		7.5	
tail/spinal malformations	0.5	1	5.56	8.89	8.53	-	10	2.96	33.3	30	
hemorrhage		1	5.56	2.96	8.53	10	10	0.99		7.5	
EC_50_ (mg/L, nominal) (cumulative sublethal end points)	0.48	1.07	3.45	1.28	2.77	5.4	6.06	0.35	6.84	17.2	283
TI	1.8	1.5	0.92	3.3	1.8	1.3	2.3	48.7	2.9	2.0	NA

a Lowest-observed effect concentrations (LOECs, mg/L) are reported for each developmental end point when at least 10% of embryos were affected in a concentration- and/or time-dependent manner. EC_50_ values for malformations were derived from the concentration–response curves established at the end of the 96 h exposure.

b Denotes parameters not included in the teratogenicity calculation (i.e., pigmentation, spontaneous movement, and blood circulation). The teratogenic index (TI) was calculated for each bisphenol as the ratio LC_50_/EC_50_, using LC_50_ values obtained in the refined FET assay. All concentrations are nominal.

To assess the teratogenic potential of the tested bisphenols, we calculated the teratogenic index (TI) based on the LC_50_ and malformation EC_50_ values. Numerous studies have reported zebrafish embryos as a useful model to identify (pro)­teratogenic compounds and to predict potential outcomes in mammals using this TI calculation,^44–46^ although different thresholds and calculation methods have been proposed in the literature, notably by including or not some specific end points, thereby reflecting a lack of harmonization for TI calculation. This approach also aligns with current efforts to assess the specificity of early life stage fish toxicity responses in order to improve environmental risk assessment.^47^ In the present study, all bisphenols were characterized by a TI > 1, except BPS-MPE (Table 3). For most bisphenols (TCBPA, BPAF, BPC, BPB, and BPF), the TI values varied between 1 and 2 (1.8, 1.5, 1.8, 1.3, and 2.0, respectively), indicating a limited teratogenic potential for these compounds. Four bisphenols showed higher TI values: BPA, BPS-MAE, and BPC-Cl had TI values of 2.3, 2.9, and 3.3, respectively, while 4,4’-ODP was characterized by a very high TI of 48.7. These results highlight the teratogenic potential of these bisphenols, thus contributing to a better characterization of the hazard assessment of these substances, with particular attention to 4,4’-ODP.

While some authors suggested the possibility of assigning molecular pathways and modes of action according to the promoted phenotypic responses and morphological fingerprints,^44,48^ we chose to perform a hierarchical clustering approach based on observed phenotypic end points to better characterize the diversity and potential similarities in the developmental toxicity profiles elicited by bisphenol substitutes (Figure 2). In line with Michaelis et al.,^48^ we expressed each end point as the percentage of affected embryos for each substance within a common concentration range. This range was defined between the lowest tested concentration at which at least 10% of embryos exhibited any phenotypic effect and the highest nonlethal concentration, in order to better capture specific developmental effects. Moreover, since the timing of appearance of end points varied during development (*e.g.*, deformity of the yolk at early developmental stages), we selected the maximum observed percentage across time points. The heatmap shows distinct phenotypic fingerprints among bisphenols, and the hierarchical clustering highlights a clear discrimination between bisphenols inducing multiphenotypic effects (i.e., 4,4’-ODP, BPF, BPC-Cl and BPA) and others inducing few morphological effects (Figure 2). Interestingly, the cluster with multiphenotypic fingerprint includes 4,4’-ODP, BPF, BPC-Cl, and BPA, which are among the test compounds having the highest TI values. The clustering also groups BPS and BPS-MAE together, both inducing a marked effect on the yolk sac.

**Figure 2:**
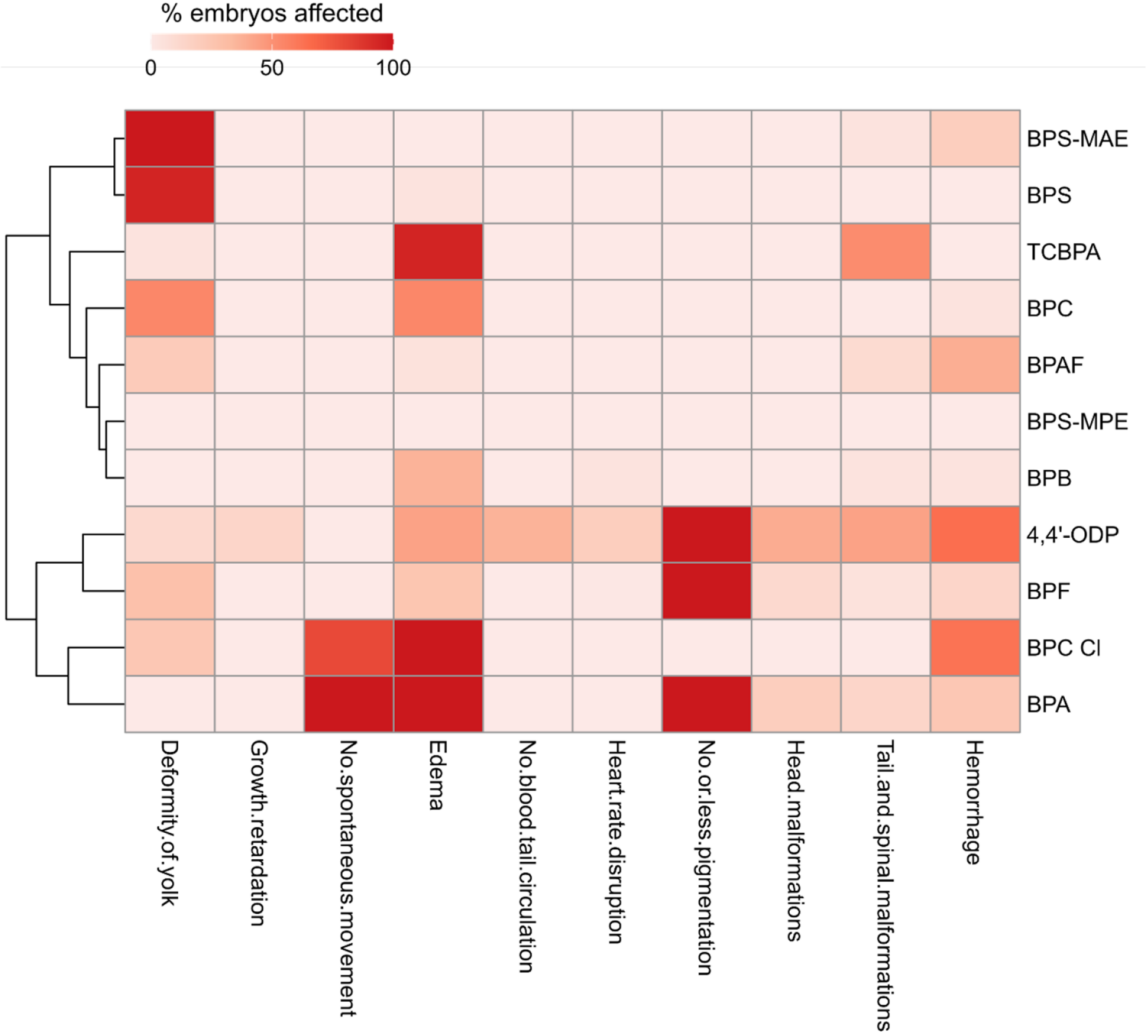
Hierarchical clustering and heatmap of phenotypic fingerprints induced by bisphenols in zebrafish embryos. The heatmap shows the percentage of affected embryos for each bisphenol and developmental end point, based on the time point with the highest response. Concentration ranges from the first concentration inducing >10% effect up to the highest nonlethal concentration were considered. Hierarchical clustering of end points was performed using Ward’s method with Euclidean distance.

### Almost All Bisphenols Can Induce GFP in *tg*(*cyp19a1b:GFP*) Zebrafish Embryos

We took advantage of using *tg*(*cyp19a1b:GFP*) embryos in the refined FET assay to image GFP in hatched embryos and gain preliminary information about the potential estrogenic activity of the tested bisphenols. Significant inductions of GFP were reported for all the bisphenols, with the notable exception of TCBPA, for which a 2-fold GFP increase was noted at 0.25 mg/L but not at the higher concentrations (Figure 3). In most cases, the induction profiles did not allow us to model a concentration-dependent response curve and derive EC_50_ values to quantify their estrogenic activity, except for BPS, for which a full concentration–response curve was obtained (EC_50_ = 35.7 mg/L; Figure S4). These data, therefore, indicate that most bisphenols elicit an estrogenic activity in the brain, which was further investigated using the EASZY assay,^14^ specifically designed to detect and quantify the estrogenic activity of test chemicals.

**Figure 3:**
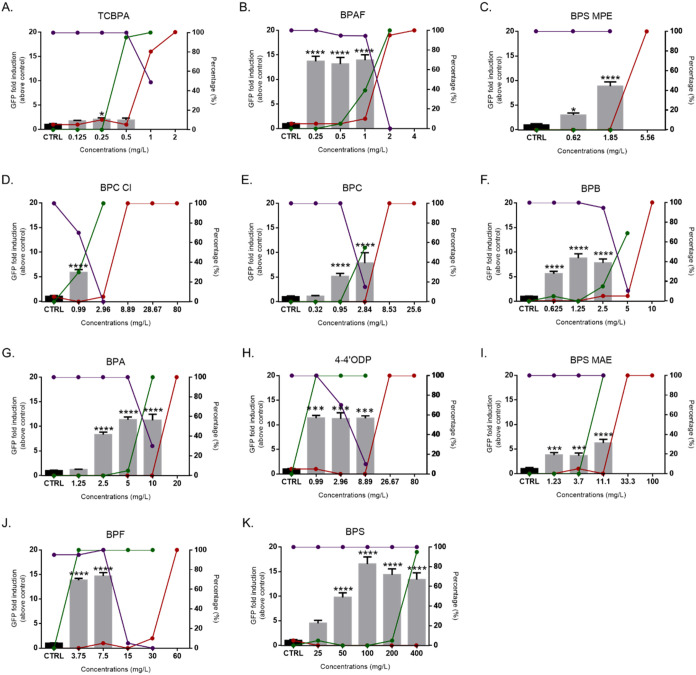
Summary of the refined FET test: GFP induction, acute toxicity, and developmental effects in zebrafish embryos exposed to bisphenols. Bar plots show GFP fold induction relative to the control group (CTRL = water or solvent control) after 96h of exposure. Red lines represent the percentage of mortality, green lines represent the percentage of embryos affected during the exposure, and violet lines represent the percentage of hatching after 96h of exposure. The left *y*-axis corresponds to GFP fold induction, and the right *y*-axis to percentages of mortality, malformations, and hatching. Asterisks denote significant differences compared with the control group (*p < 0.05; ***p < 0.0005; ****p < 0.0001). Data are represented as mean ± SEM. All concentrations are nominal. Labels from A−K refer to the various bisphenols tested, with their abbreviations listed in the figure.

### In the EASZY Assay, All Bisphenols Except BPS Showed Higher Estrogenic Activity than BPA

Based on the preliminary data obtained from the FET, we refined the concentrations to be used in the EASZY assay (Figure 3) to obtain accurate information about the estrogenic activity of bisphenols. The maximum concentrations to be tested were based on the following requirements: they should not (i) induce mortality, (ii) affect the hatching rate (Figure S2), or (iii) significantly induce developmental effects in zebrafish embryos. Results obtained from the EASZY assays confirmed the absence of estrogenic activity for TCBPA in zebrafish embryos. Interestingly, the absence of estrogenic activity of TCBPA found in *tg*(*cyp19a1b:GFP*) embryos in the refined FET and the EASZY assays further supports the data found in adult zebrafish using measurements of circulating vitellogenin concentrations as an ER-specific end point.^25^ Furthermore, the results from the EASZY assay confirmed the estrogenic activity of all other bisphenols, as shown by the concentration-dependent induction of GFP in the developing brain of zebrafish (Figure 4). Quantitative analysis of GFP fluorescence further revealed distinct induction profiles among the compounds (Figure 5). Some bisphenols (i.e., 4,4’-ODP, BPF, and BPS) induced full concentration-dependent response curves with maximal responses reaching the GFP levels measured in the positive control (i.e., EE2 14.8 ng/L), while other bisphenols were characterized by maximal fold-induction levels below GFP levels measured in the positive control. For BPS-MAE, significant inductions were measured from 0.625 mg/L, but an unusual concentration–response curve was observed as the response reached a plateau at low induction levels (Figures 5I; S5). The observed differences in induction profiles could be explained, at least partially, by differences in the agonist activity of bisphenols toward zebrafish ERs (see below). Based on these induction profiles, the relative ECx values (Table 4) were derived from the complete modeled concentration–responses curves for GFP (Figure S5), in order to quantify the estrogenic activity of each bisphenol and allow for ranking them from the less active to the most active. Based on their EC_50_ values, the bisphenols were ranked as follows: BPS ≪ BPA < BPS-MPE < BPC ∼ BPB < BPF < 4,4’-ODP < BPS-MAE ≪ BPAF ≪ BPC-Cl, with “∼” indicating <10%, “<” indicating 10–100%, and “≪” indicating >100% differences.

**Figure 4:**
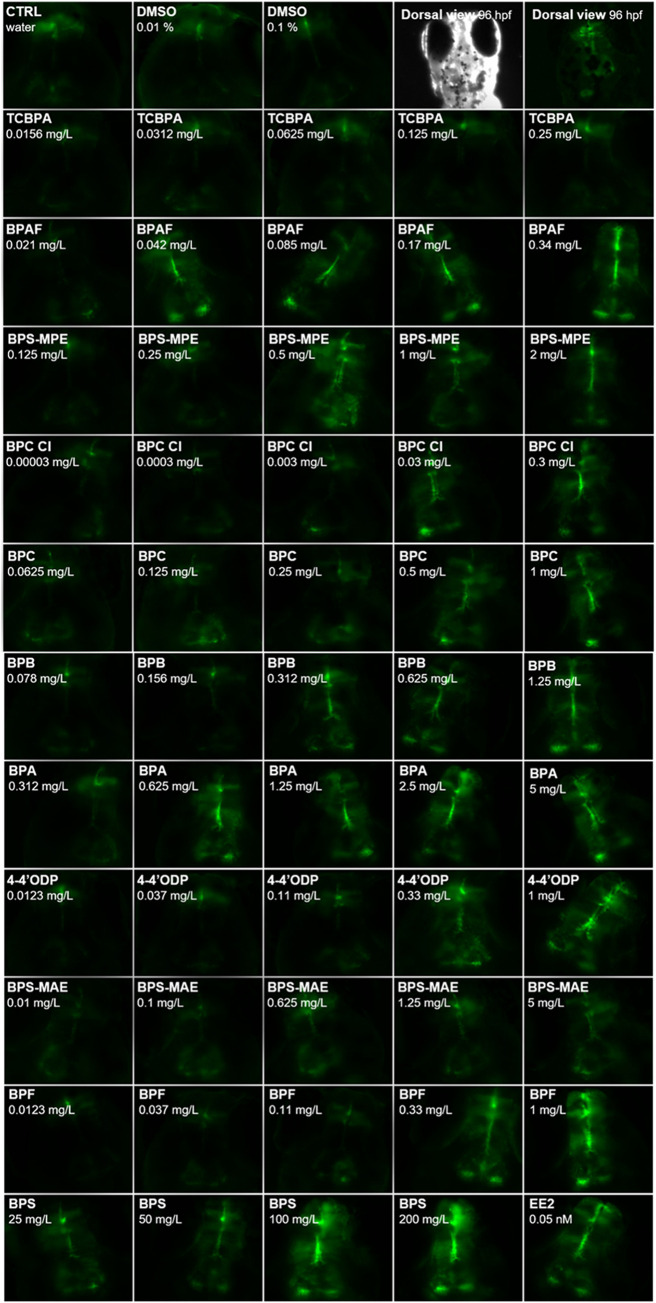
Representative fluorescence images of *tg*(*cyp19a1b:GFP*) zebrafish embryos exposed to bisphenols (96 h of exposure, dorsal views of the head). Images illustrate GFP induction in radial glial cells following exposure to increasing concentrations of each bisphenol. For each chemical, the nominal concentration used is indicated. CTRL = water control; DMSO = solvent control; EE2 = positive control, 0.05 nM. In the top row of the figure, a brightfield image of a zebrafish embryo exposed to DMSO 0.01% is shown, with its corresponding fluorescence image, to illustrate the positioning of each embryo for GFP imaging.

**Figure 5:**
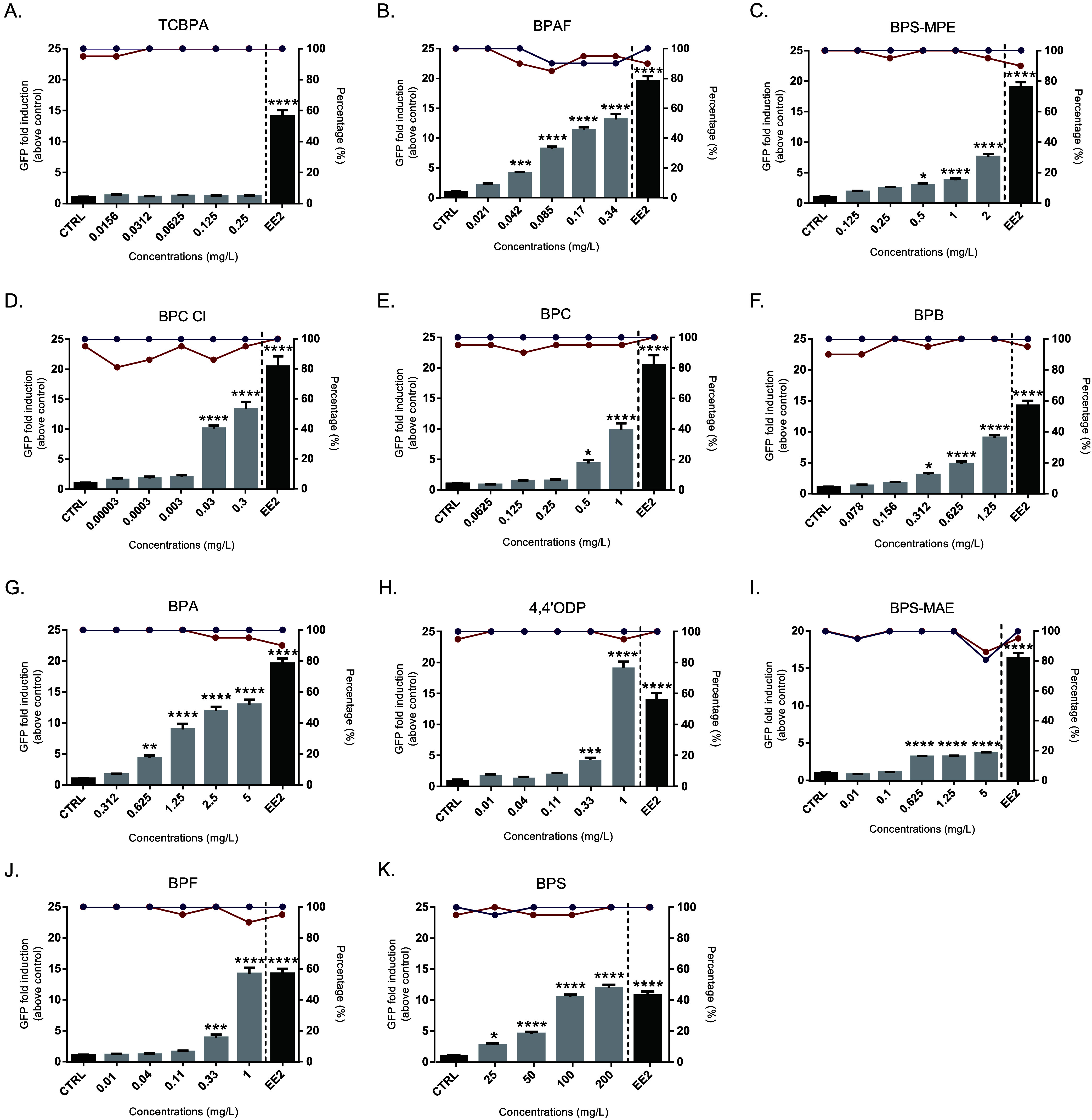
Summary of the EASZY assay: GFP induction, survival, and hatching in *tg*(*cyp19a1b:GFP*) zebrafish embryos exposed to bisphenols. Bar plots show GFP fold induction (mean ± SEM) relative to the solvent control (CTRL) after 96 h of exposure. Red lines indicate survival percentage, and violet lines indicate hatched embryo percentage after 96 h of exposure. The left *y*-axis corresponds to GFP fold induction, and the right *y*-axis to percentages of survival and hatching. Asterisks denote significant differences compared with the control group (*p < 0.05; ***p < 0.0005; ****p < 0.0001). EE2 concentration = 0.05 nM/14.8 ng/L. See Figure S5 for complete modeled curves. All concentrations are nominal. Labels from A−K refer to the various bisphenols tested, with their abbreviations listed in the figure.

**Table 4:** Estrogenic Activity of Bisphenols in Zebrafish Embryos: Relative ECx Values from EASZY (Nominal Concentrations), Maximal Fold Induction, and Sensitivity Ratios^a^

chemicals	EC_10_ (mg/L)	EC_20_ (mg/L)	EC_50_ (mg/L)	max fold	max fold EE2	sensitivity ratio (SR) (LC_10_/EC_10_)
TCBPA	n.a.	n.a.	n.a.	n.a.	14.8	n.a.
BPAF	0.022	0.035	0.075	13.1	19.6	50.9
BPS-MPE	0.186	0.317	0.792	7.6	18.9	6.36
BPC-Cl	0.0031	0.0055	0.0149	13.4	20.4	826
BPC	0.37	0.425	0.538	9.8	20.4	9.61
BPB	0.196	0.283	0.529	9.0	14.2	20.9
BPA	0.358	0.519	0.98	12.9	19.5	26.2
4,4’-ODP	0.315	0.332	0.363	19.2	14.2	47.9
BPS-MAE	0.105	0.16	0.327	3.6	16.3	45.9
BPF	0.278	0.32	0.407	14.2	14.2	111
BPS	26.36	36.29	62.69	11.9	10.7	n.a.

a Data are expressed in mg/L (nominal concentrations). ECx values correspond to relative ECx (normalized to the modeled upper asymptote; see Figure S5 for complete modeled curves). Max fold indicates the mean maximum fold induction measured for each bisphenol. Max fold EE2 corresponds to the mean fold induction of the 0.05 nM EE2 positive control. The sensitivity ratio (SR) was calculated as the ratio between acute toxicity LC_10_ (FET assay) and estrogenicity absolute EC_10_, also referred to as PC_10_ (SR = LC_10_/absolute EC_10_).

The estrogenic activity of BPC-Cl, BPAF, BPS-MAE, 4,4’-ODP, BPF, BPB, BPC, and BPS-MPE were, respectively, 65, 13, 3, 2.7, 2.45, 1.85, 1.8, and 1.25-fold higher than BPA (EC_50_ = 0.98). Among all the bisphenols tested, BPS was the only one less active than BPA. Overall, our study confirms the estrogenic activity of several bisphenols (i.e., BPA, BPAF, BPB, BPC, BPC-Cl, BPF, BPS) on different zebrafish ER-sensitive embryo models,^9,29,40,49–52^ and provides new quantitative data concerning their estrogenic activity. In addition, to our knowledge, our study also reports the first quantitative *in vivo* estrogenic activity for three additional BPA substitutes (4,4’-ODP, BPS-MAE, BPS-MPE), all of them being characterized by a higher estrogenic activity than BPA.

The sensitivity ratios (SR: LC_10_/EC_10_), commonly used in *in vitro* reporter gene assays^53–55^ and in *in vivo* zebrafish embryos assays,^56,57^ were calculated to evaluate the specificity of estrogenic activity of bisphenols in zebrafish embryos. All tested bisphenols displayed SR values ranging from 6.4 to 826 (Table 4), indicating that estrogenic activity occurred at concentrations below those causing acute toxicity, consistent with a specific mode of action of these compounds on the ER signaling pathway. For most of the bisphenol substitutes tested, the SR was greater than the TI, indicating also a specificity as compared to developmental effects, except for 4,4’-ODP for which the SR was equal to the TI, due to the induction of hemorrhages concomitantly with its estrogenic activity (without interfering with the *in vivo* imaging). For BPS, no SR could be determined due to the absence of acute toxicity at the tested concentrations.

### Bisphenols Act as Agonists of zfERs, Which Are Required to Induce Brain Aromatase In Vivo

We further studied the capacity of bisphenols to interact with the ER signaling pathway at the molecular level by performing a set of *in vivo* and *in vitro* experiments. We first performed coexposure experiments of embryos with the ER antagonist ICI at 1 μM (607 μg/L) and one concentration of each bisphenol, i.e., the lowest nominal concentration leading to the maximal induction of GFP. The induction of GFP expression by bisphenols was partially, but not totally, downregulated in the presence of ICI (Figure S6), showing that functional ERs are required in the induction of the brain aromatase gene by these compounds, as previously demonstrated for some bisphenols.^9,49,52^ However, by using this approach, ICI 1 μM was unable to block the effect of BPC-Cl and BPS-MAE, even when zebrafish embryos were pre-exposed to ICI alone during the first 48 h (data not shown). The reason for this lack of effect of ICI is not known. It could simply reflect nonoptimal coexposure conditions unable to efficiently block the effects of bisphenols, especially those with a high estrogenic activity such as BPC-Cl. Indeed, Kubota et al.^52^ reported that higher ICI concentrations were able to fully inhibit their effects on the expression of the endogenous *cyp19a1b* gene in zebrafish embryos.

We also further characterized the mode of action of bisphenols on ER signaling by studying the transactivation of zebrafish ERβ2 in the specific ZELH-zfERβ2 cell line.^15^ This cell line was selected because of its higher sensitivity to natural and synthetic steroidal estrogens, including E2, estrone, estriol, and 17α-ethinylestradiol,^15^ and to xeno-estrogens such as BPF and BPS,^9^ and its higher responsiveness to environmental extracts^58^ compared to the ZELH-zfERα cell line. Furthermore, zfERβ2 is one of the first zfER isoforms to be expressed in the zebrafish brain at early stages of development,^16^ hence reinforcing the relevance of assessing this receptor in the present study. The results showed that, except BPS-MAE and BPS-MPE (Table 5; Figure S7), all of the tested bisphenols induced luciferase activity in the presence of zebrafish ERβ2 (Table 5; Figure S7). No induction of luciferase was observed at the test concentrations in the ZELH cell line that expresses no functional ER (data not shown), suggesting that the effects observed in ZELH-zfERβ2 cells were receptor-specific. For active bisphenols, EC_50_/PC_50_ values, which ranged from 0.05 to 9.6 μM (Table 5), were of the same order as those reported in human *in vitro* reporter cell lines.^55,59,60^ Four out of the 11 bisphenols had lower EC_50_ compared to BPA, BPC-Cl being the most potent zfERβ2 agonist with a 26-fold higher estrogenic activity compared to BPA, which is consistent with a previous report in human HELN ERα and ERβ cell line.^59^ Based on EC_50_ values, some differences were also noticed when comparing zebrafish and human cell-based responses. For instance, BPAF appeared less active on zfERβ2 than on hERβ and hERα.^55,59,60^ Conversely, BPF and 4,4’-ODP were found to be much more active on zfERβ2 with 2- and 8.7-fold higher estrogenic activity as compared to BPA, while they have been reported to be less active than BPA on hERβ and hERα.^55,59^

**Table 5:** *In Vitro* Zebrafish Estrogen Receptor β2 Transactivation by Bisphenols: Relative EC_50_ or PC_50_ Values and Maximal Efficiency in ZELH-zfERβ2 Cells Compared With *In Vitro* Human ERβ Reporter Gene Assays from the Literature[Table-fn t5fn1]

	ZELH-zfERβ2 (this study)			HELN-hERβ Grimaldi et al.^59^		HepG2-hERβ Pelch et al.^60^	
chemicals	EC_50_ (μM)	PC50 (μM)	maximal efficacy (%)	EC_50_ (μM)	maximal efficacy (%)	EC_50_ (μM)	maximal efficacy (%)
E2	0.00006			0.00009		0.0011	
BPC-Cl	0.05		61%	0.046	35%	NT	
4,4′-ODP	0.15		61%	3.06	72%	NT	
BPC	0.42		66%	0.559	31%	3.2	75%
BPF	0.67		71%	0.952	100%	NT	
BPA	1.36		55%	0.384	66%	0.35	97%
BPB		2.57	66%	0.128	60%	NC	56%
BPS		4.20	85%	1.72	100%	2.1	77%
TCBPA	5.06		42%	68	21%	NC	15%
BPAF		9.60	46%	0.068	71%	0.046	85%
BPS-MPE	NA			NT		NA	4%
BPS-MAE	NA			NT		NA	6%

1 Relative EC_50_ (μM), absolute EC_50_ (also referred as PC_50_: % of the positive control response), and maximal transactivation efficacy (%) are reported for each bisphenol in the ZELH-zfERβ2 cell line and compared with two published human Erβ reporter gene assays. NA = not active; NT = not tested; NC = not calculated. See Figure S7 for complete modeled curves. All concentrations are nominal.

Overall, active bisphenols showed partial efficiency to transactivate zfERβ2, reaching 42–85% of the maximum activity induced by E2 (Table 5; Figure S7). This agrees with the already described partial agonist activity of BPA and bisphenol substitutes.^49,55,59–61^ Nevertheless, some differences with human models can still be noticed, since BPA, BPF, or BPS were shown to act as full agonist ligands depending on the cell system used.^59,60^ Such differences in activity and potency depending on the cell context support the view that BPA and BPA analogues can act as selective ER modulators (SERM) in mammals and fish, which may account for the tissue-specific responses observed *in vivo*.^9,29,49^

Altogether, these experiments provide further evidence that most bisphenols act as agonist ligands of zebrafish ERs, which are required to upregulate *cyp19a1b* brain aromatase expression in developing zebrafish in a concentration-dependent manner.

### Strengths and Limits of the Combined Refined FET and EASZY Assays

This study shows that the combination of the two zebrafish-embryo OECD TGs (TG N°236 and TG N°250) using *tg*(*cyp19a1b:GFP*) zebrafish embryos as a common model allowed us to obtain complementary information on the toxicity, developmental effects, and estrogenic activity of chemicals. Using the same transgenic embryo model in both assays ensures consistency in determining sublethal concentrations to be used in EASZY assay and provides preliminary information on the estrogenic potency of test chemicals. This approach can guide the decision on whether the EASZY assay is further required, potentially reducing unnecessary testing in line with the 3R principles. Applied to 11 bisphenols, it efficiently allows us to collect toxicological information for these compounds (see results compiled in SI Data set S1), thereby providing new and relevant data for their hazard assessment. It also provides a simple and systematic workflow to generate robust, reliable, and regulatory-relevant data that would serve for the assessment of chemicals for which hazard information is lacking. As compared to acute and developmental toxicity data, which are becoming more available, information on endocrine-disrupting properties remains limited, despite the risks they pose and the regulatory needs and challenges identified by ECHA.^62^ The proposed methodological approach based on the use of the *tg*(*cyp19a1b:GFP)* model for estrogenic activity faithfully meets the regulatory needs for xenoestrogens. Based on this simple conceptual and methodological approach, it is easily conceivable to use other transgenic zebrafish models allowing the identification of chemicals acting through estrogen, androgen, thyroid, and steroidogenesis pathways (EATS) as well as non-EATS modes of action. For instance, some transgenic zebrafish embryo models have been developed and proven reliable for detecting chemicals acting on the thyroid axis (*e.g., tg­(thyroglobulin:mcherry)*)^63^ or the metabolism (*e.g., tg*(*cyp3a65:GFP*)).^64^ Notwithstanding, it is important to ensure that the sensitivity of these transgenic models to toxic chemicals is documented.

When comparing the *in vivo* and *in vitro* data, we found overall good agreement in estrogenic activity, both qualitatively and quantitatively. For most bisphenols, the difference between *in vivo* and *in vitro* effective concentrations remained within a factor of 5, indicating a consistent response across test systems. However, there was a noticeable difference observed for BPS, which was much less active in embryos than *in vitro*, as previously reported in the literature.^9^ This difference in bioactivity reflects the particular toxicokinetic profile of BPS in zebrafish embryos compared to its congeners,^65^ which is characterized by low bioavailability and rapid metabolism into inactive metabolites.^66^ Interestingly, TCBPA was inactive in embryos while weakly active in zebrafish and human cell lines,^55,59^ but at concentrations that were toxic to zebrafish embryos, suggesting its environmental hazard relies more on its acute toxicity rather than on its estrogenic activity. It is also critical to emphasize that BPS-MAE and BPS-MPE were identified as inactive in fish cell-based assays in this study, but also in human cell-based assays in the literature.^60^ Interestingly, a recent study confirmed the lack of activity of BPS-MPE toward hERα *in vitro*, while BPS-MAE was found to be weakly active only at high concentrations.^55^ In contrast, BPS-MAE and BPS-MPE were clearly active *in vivo*, inducing estrogenic activity in *tg*(*cyp19a1:GFP*) embryos. It is now well-known that zebrafish embryos are metabolically competent, having biotransformation capacities catalyzing both Phase I and Phase II enzymatic reactions.^66–68^ Furthermore, previously published data have shown that the EASZY assay may also provide information on the estrogenic activity of pro-estrogenic chemicals, i.e., those that require metabolic activation prior to eliciting an estrogenic response.^19,69^ The metabolic activities of zebrafish embryos as compared to those of cell-based assays may explain why these two bisphenols induce an estrogenic response *in vivo* but not *in vitro*. Interestingly, the influence of CYP-mediated metabolic activation of bisphenols and the resulting estrogenic activity was recently evaluated *in vitro*.^55^ Using abiotic CYP oxidation, the authors did not observe any increase in the estrogenic activity of BPS-MAE and BPS-MPE, suggesting that the *in vivo* effect observed in EASZY may not be driven by the phase I metabolism. Another hypothesis to explain the brain estrogenic activity of BPS-MAE and BPS-MPE *in vivo* could rely on the fact that these substances may act as SERMs, as they induce estrogenic activity in a radial glial cell context but not in hepatic cells. Overall, the differential estrogenic activity between *in vitro* ER transactivation assays and the *in vivo* EASZY assay reported herein for the first time calls for further investigation through specific metabolism and toxicokinetic studies and highlights the importance of considering whole organism responses to reliably inform on the toxicity and endocrine activity of test chemicals, as done in the present work.

As previously mentioned, EASZY is the only OECD test guideline capable of informing on the (neuro)­endocrine activity of test chemicals occurring in radial glial cells (RGCs) in a vertebrate model, but one should keep in mind that there is a gap between quantification of estrogen-like activities using EASZY and informed risk assessment. Establishing causal and possibly quantitative links between disruption of the brain aromatase gene during early brain development and adverse health effects in fish is a current research challenge. In mammals and fish, RGCs behave as neural stem cells during embryonic development, but in contrast to mammals, RGCs persist in fish throughout the entire lifespan, supporting the high neurogenic activity observed in adult fish.^70^ There is evidence that estrogens regulate neurogenesis, proliferation, and apoptosis in the zebrafish brain,^71,72^ brain aromatase being responsible for neuroestrogen synthesis. Furthermore, embryonic exposure to chemicals known to act on brain aromatase has been shown to alter neurogenesis and locomotor behavior in zebrafish embryos,^40,73,74^ and even lead to long-term behavioral responses in adults.^75^ However, it is difficult to establish a clear link between brain aromatase modulation and specific behavioral phenotypic responses, given that substances capable of inducing or inhibiting brain aromatase expression in the developing brain can also interact with other molecular and cellular targets other than ERs and brain aromatase, highlighting the need to further explore the mechanisms of chemicals on behavioral outcomes. Nevertheless, evidence from studies using pharmacological approaches and brain aromatase mutants in adult zebrafish supports the involvement of brain aromatase expression and/or activity in neuroplasticity, social behavior, aggressiveness, and locomotion in adult fish.^76,77^ Altogether, these studies show that brain aromatase plays a critical role in brain development and behavioral responses in zebrafish, suggesting that its modulation by chemicals could have direct adverse effects on exposed individuals.

Overall, this study illustrates the relevance of combining two OECD test guidelines (TG 236 and TG 250) within the same transgenic zebrafish model to efficiently inform on the acute toxicity, developmental effects, and estrogenic activity of BPA and some of its substitutes. The proposed methodological approach significantly contributes to the improvement of the environmental hazard assessment of chemicals by generating harmonized data of significant relevance for environmental toxicologists, industry, and risk assessors and argues for additional studies on data-poor chemicals.

## Supplementary Material





## Notes

All data generated in this study are compiled in data set S1 (XLSX)
Nominal concentrations of tested chemicals used in the FET tests (Table S1); nominal concentrations of tested chemicals used in the EASZY assays (Table S2); summary of toxic responses and malformations from the literature and this study (Table S3); concentration–response curves for mortality in the FET test (Figure S1); hatching success across time and concentrations (Figure S2); representative malformation types observed in the FET tests (Figure S3); concentration–response curves of cyp19a1b-driven GFP for BPS in the FET tests (Figure S4); concentration–response curves of cyp19a1b-driven GFP in EASZY assays (Figure S5); cyp19a1b-driven GFP induction after coexposure with ICI 182,780 (Figure S6); concentration–response curves of luciferase in the ZELH-zfERβ2 cells (Figure S7) (PDF)

## ∇

Université de Rennes, CNRS, Normandie Université, EthoS (Éthologie animale et humaine), UMR 6552, F-35000 Rennes, France

## Abbreviations

OECD: Organisation for Economic Co-operation and Development
FET: Fish Embryo Acute Toxicity
EASZY: detection of endocrine active substances, acting through estrogen receptors, using transgenic tg­(cyp19a1b:GFP) Zebrafish embrYos
GFP: green fluorescent protein
BPA: bisphenol A
BPF: bisphenol F
BPS: bisphenol S
BPB: bisphenol B
BPAF: bisphenol AF
BPS-MAE: bisphenol S monoallyl ether
BPS-MPE: bisphenol S monopropyl ether
TCBPA: tetrachloro-bisphenol A
BPC: bisphenol C
BPC-Cl: bisphenol C chlorinated analogue
DMSO: dimethyl sulfoxide
EE2: 17α-ethinylestradiol
4,4′-ODP: 4,4′-oxydiphenol
EDC: endocrine disrupting chemical
ER: estrogen receptor
LCx: lethal concentration inducing x% mortality
ECx: effective concentration inducing x% effect
LOEC: lowest observed effect concentration
TI: teratogenic index
TR: toxic ratio
SR: sensitivity ratio
dpf: days post fertilization
hpf: hours post fertilization

## References

[1] Oehlmann J., Schulte-Oehlmann U., Kloas W., Jagnytsch O., Lutz I., Kusk K. O., Wollenberger L., Santos E. M., Paull G. C., Look K. J. W. V., Tyler C. R. (2009). A critical analysis of the biological impacts of plasticizers
on wildlife. Philos. Trans. R. Soc., B.

[2] Rochester J. R. (2013). Bisphenol
A and human health: a review of the literature. Reprod. Toxicol..

[3] Lambré C., Barat Baviera J. M., Bolognesi C., Chesson A., Cocconcelli P. S., Crebelli R., Gott D. M., Grob K., Lampi E., Mengelers M., Mortensen A., Rivière G., Silano V., Steffensen I.-L., Tlustos C., Vernis L., Zorn H., Batke M., Bignami M., Corsini E., FitzGerald R., Gundert-Remy U., Halldorsson T., Hart A., Ntzani E., Scanziani E., Schroeder H., Ulbrich B., Waalkens-Berendsen D., Woelfle D., Al Harraq Z., Baert K., Carfì M., Castoldi A. F., Croera C., Van Loveren H., EFSA Panel on Food Contact Materials, Enzymes and Processing Aids
(CEP) (2023). Re-Evaluation of the
Risks to Public Health Related to the Presence of Bisphenol a (BPA)
in Foodstuffs. EFSA J..

[4] Regulation - EU - 2024/3190 - EN - EUR-Lex. https://eur-lex.europa.eu/eli/reg/2024/3190/oj/eng (accessed Sept 2, 2025).

[5] Chen D., Kannan K., Tan H., Zheng Z., Feng Y.-L., Wu Y., Widelka M. (2016). Bisphenol
analogues other than BPA: environmental occurrence,
human exposure, and toxicity—a review. Environ. Sci. Technol..

[6] Sun Q., Wang Y., Li Y., Ashfaq M., Dai L., Xie X., Yu C.-P. (2017). Fate and
mass balance of bisphenol analogues in wastewater
treatment plants in Xiamen city, China. Environ.
Pollut..

[7] Yu X., Xue J., Yao H., Wu Q., Venkatesan A. K., Halden R. U., Kannan K. (2015). Occurrence
and estrogenic potency
of eight bisphenol analogs in sewage sludge from the U.S. EPA targeted
national sewage sludge survey. J. Hazard. Mater..

[8] Adamovsky O., Groh K. J., Białk-Bielińska A., Escher B. I., Beaudouin R., Mora Lagares L., Tollefsen K. E., Fenske M., Mulkiewicz E., Creusot N., Sosnowska A., Loureiro S., Beyer J., Repetto G., Štern A., Lopes I., Monteiro M., Zikova-Kloas A., Eleršek T., Vračko M., Zdybel S., Puzyn T., Koczur W., Ebsen Morthorst J., Holbech H., Carlsson G., Örn S., Herrero Ó., Siddique A., Liess M., Braun G., Srebny V., Žegura B., Hinfray N., Brion F., Knapen D., Vandeputte E., Stinckens E., Vergauwen L., Behrendt L., João Silva M., Blaha L., Kyriakopoulou K. (2024). Exploring BPA alternatives –
environmental levels and toxicity review. Environ.
Int..

[9] Le
Fol V., Aït-Aïssa S., Sonavane M., Porcher J.-M., Balaguer P., Cravedi J.-P., Zalko D., Brion F. (2017). *In
Vitro* and *in Vivo* Estrogenic Activity of
BPA, BPF and BPS in zebrafish-specific assays. Ecotoxicol. Environ. Saf..

[10] Molina-Molina J.-M., Amaya E., Grimaldi M., Sáenz J.-M., Real M., Fernández M. F., Balaguer P., Olea N. (2013). In vitro study
on the agonistic and antagonistic activities of bisphenol-S and other
bisphenol-A congeners and derivatives via nuclear receptors. Toxicol. Appl. Pharmacol..

[11] Stanojević M., Sollner Dolenc M. (2025). Mechanisms
of bisphenol A and its analogs as endocrine
disruptors via nuclear receptors and related signaling pathways. Arch. Toxicol..

[12] Serra H., Beausoleil C., Habert R., Minier C., Picard-Hagen N., Michel C. (2019). Evidence for bisphenol B endocrine properties: scientific
and regulatory perspectives. Environ. Health
Perspect..

[13] OECD . Test No. 236: Fish Embryo Acute Toxicity (FET) Test, OECD Guidelines for the Testing of Chemicals, Section 2; OECD Publishing: Paris, 2025, 10.1787/9789264203709-en.

[14] OECD . Test No. 250: EASZY assay - Detection of Endocrine Active Substances, acting through estrogen receptors, using transgenic tg(cyp19a1b:GFP) Zebrafish embrYos, OECD Guidelines for the Testing of Chemicals, Section 2; OECD Publishing; Paris, 2021, 10.1787/0a39b48b-en.

[15] Cosnefroy A., Brion F., Maillot-Maréchal E., Porcher J.-M., Pakdel F., Balaguer P., Aït-Aïssa S. (2012). Selective
activation of zebrafish estrogen receptor subtypes by chemicals by
using stable reporter gene assay developed in a zebrafish liver cell
line. Toxicol. Sci..

[16] Mouriec K., Lareyre J., Tong S., Le Page Y., Vaillant C., Pellegrini E., Pakdel F., Chung B., Kah O., Anglade I. (2009). Early regulation
of brain aromatase (*cyp19a1b*) by estrogen receptors
during zebrafish development. Dev. Dyn..

[17] Tong S.-K., Mouriec K., Kuo M.-W., Pellegrini E., Gueguen M.-M., Brion F., Kah O., Chung B. (2009). A cyp19a1b-GFP
(aromatase b) transgenic zebrafish line that expresses GFP in radial
glial cells. Genesis.

[18] ISO . ISO 6341. https://www.iso.org/standard/54614.html (accessed Nov 5, 2025).

[19] Brion F., Le Page Y., Piccini B., Cardoso O., Tong S.-K., Chung B., Kah O. (2012). Screening
estrogenic activities of
chemicals or mixtures in vivo using transgenic (cyp19a1b-gfp) zebrafish
embryos. PLoS One.

[20] Ritz C., Baty F., Streibig J. C., Gerhard D. (2015). Dose-Response Analysis
Using R. PLoS One.

[21] Team, R. R. A language and environment for statistical computing. MSOR Connections, 2014.

[22] Klüver N., Vogs C., Altenburger R., Escher B. I., Scholz S. (2016). Development
of a general baseline toxicity QSAR model for the fish embryo acute
toxicity test. Chemosphere.

[23] Kolde, R. Pheatmap: Pretty Heatmaps. R Package, 2019.

[24] Busquet F., Strecker R., Rawlings J. M., Belanger S. E., Braunbeck T., Carr G. J., Cenijn P., Fochtman P., Gourmelon A., Hübler N., Kleensang A., Knöbel M., Kussatz C., Legler J., Lillicrap A., Martínez-Jerónimo F., Polleichtner C., Rzodeczko H., Salinas E., Schneider K. E., Scholz S., van den Brandhof E.-J., van der Ven L. T. M., Walter-Rohde S., Weigt S., Witters H., Halder M. (2014). OECD validation
study to assess intra- and inter-laboratory reproducibility of the
zebrafish embryo toxicity test for acute aquatic toxicity testing. Regul. Toxicol. Pharmacol..

[25] Song M., Liang D., Liang Y., Chen M., Wang F., Wang H., Jiang G. (2014). Assessing
developmental toxicity
and estrogenic activity of halogenated bisphenol A on zebrafish (Danio
Rerio). Chemosphere.

[26] Arrokhman S., Luo Y.-H., Lin P. (2023). Additive cardiotoxicity
of a bisphenol
mixture in zebrafish embryos: the involvement of calcium channel and
pump. Ecotoxicol. Environ. Saf..

[27] Ren W.-J., Wang Z., Yang X., Liu J., Yang Y., Chen Y., Shen S. (2017). Acute toxicity effect
of bisphenol
A and its analogues on adult and embryo of zebrafish. J. Ecol. Rural Environ..

[28] Mu X., Huang Y., Li X., Lei Y., Teng M., Li X., Wang C., Li Y. (2018). Developmental
effects and estrogenicity
of bisphenol A alternatives in a zebrafish embryo model. Environ. Sci. Technol..

[29] Moreman J., Lee O., Trznadel M., David A., Kudoh T., Tyler C. R. (2017). Acute toxicity,
teratogenic, and estrogenic effects of bisphenol A and its alternative
replacements bisphenol S, bisphenol F, and bisphenol AF in zebrafish
embryo-larvae. Environ. Sci. Technol..

[30] Gao Y., Li A., Zhang W., Pang S., Liang Y., Song M. (2022). Assessing
the toxicity of bisphenol A and its six alternatives on zebrafish
embryo/larvae. Aquat. Toxicol..

[31] Blanc M., Rüegg J., Scherbak N., Keiter S. H. (2019). Environmental chemicals
differentially affect epigenetic-related mechanisms in the zebrafish
liver (ZF-L) cell line and in zebrafish embryos. Aquat. Toxicol..

[32] Chow W. S., Chan W. K., Chan K. M. (2013). Toxicity assessment
and vitellogenin
expression in zebrafish (*danio rerio*) embryos and
larvae acutely exposed to bisphenol A, Endosulfan, Heptachlor, Methoxychlor
and Tetrabromobisphenol A. J. Appl. Toxicol..

[33] Gu J., Wu J., Xu S., Zhang L., Fan D., Shi L., Wang J., Ji G. (2020). Bisphenol F exposure impairs neurodevelopment
in zebrafish larvae (*Danio Rerio*). Ecotoxicol. Environ. Saf..

[34] Gu J., Zhang J., Chen Y., Wang H., Guo M., Wang L., Wang Z., Wu S., Shi L., Gu A., Ji G. (2019). neurobehavioral effects
of bisphenol S exposure in
early life stages of zebrafish larvae (*Danio Rerio*). Chemosphere.

[35] Klüver N., Bittermann K., Escher B. I. (2019). QSAR for baseline toxicity and classification
of specific modes of action of ionizable organic chemicals in the
zebrafish embryo toxicity test. Aquat. Toxicol..

[36] Verhaar H. J. M., Urrestarazu Ramos E., Hermens J. L. M. (1996). Classifying environmental
pollutants. 2: separation of class 1 (baseline toxicity) and class
2 (‘polar narcosis’) type compounds based on chemical
descriptors. J. Chemom..

[37] Stenzel A., Wirt H., Patten A., Theodore B., King-Heiden T. (2019). Larval exposure
to environmentally relevant concentrations of triclosan impairs metamorphosis
and reproductive fitness in zebrafish. Reprod.
Toxicol..

[38] Scholz S., Schreiber R., Armitage J., Mayer P., Escher B. I., Lidzba A., Léonard M., Altenburger R. (2018). Meta-analysis
of fish early life stage tests—association of toxic ratios
and acute-to-chronic ratios with modes of action. Environ. Toxicol. Chem..

[39] Qiu W., Zhao Y., Yang M., Farajzadeh M., Pan C., Wayne N. L. (2016). Actions of bisphenol
A and bisphenol S on the reproductive
neuroendocrine system during early development in zebrafish. Endocrinology.

[40] Coumailleau P., Trempont S., Pellegrini E., Charlier T. D. (2020). Impacts of bisphenol
A analogues on zebrafish post-embryonic brain. J. Neuroendocrinol..

[41] Gajbhiye D. S., Fernandes G. L., Oz I., Nahmias Y., Golan M. (2024). A transient
neurohormonal circuit controls hatching in fish. Science.

[42] De
la Paz J. F., Beiza N., Paredes-Zúñiga S., Hoare M. S., Allende M. L. (2017). Triazole fungicides inhibit zebrafish
hatching by blocking the secretory function of hatching gland cells. Int. J. Mol. Sci..

[43] Mu X., Liu J., Yuan L., Huang Y., Qian L., Wang C. (2020). The pigmentation
interference of bisphenol F and bisphenol A. Environ. Pollut..

[44] Jarque S., Rubio-Brotons M., Ibarra J., Ordoñez V., Dyballa S., Miñana R., Terriente J. (2020). Morphometric
analysis of developing zebrafish embryos allows predicting teratogenicity
modes of action in higher vertebrates. Reprod.
Toxicol..

[45] Selderslaghs I. W. T., Van Rompay A. R., De Coen W., Witters H. E. (2009). Development
of a screening assay to identify teratogenic and embryotoxic chemicals
using the zebrafish embryo. Reprod. Toxicol..

[46] Weigt S., Huebler N., Strecker R., Braunbeck T., Broschard T. H. (2011). Zebrafish (Danio Rerio) embryos as a model for testing
proteratogens. Toxicology.

[47] Meador J. P., Escher B. I. (2025). Fish early-life
stage toxicity and environmental relevance:
what does high-dose toxicity testing tell us?. Environ. Toxicol. Chem..

[48] Michaelis P., Klüver N., Aulhorn S., Bohring H., Bumberger J., Haase K., Kuhnert T., Küster E., Krüger J., Luckenbach T., Massei R., Nerlich L., Petruschke S., Schnicke T., Schnurpel A., Scholz S., Schweiger N., Sielaff D., Busch W. (2025). Leveraging
zebrafish embryo phenotypic observations to advance data-driven analyses
in toxicology. Environ. Sci. Technol..

[49] Pinto C., Hao R., Grimaldi M., Thrikawala S., Boulahtouf A., Aït-Aïssa S., Brion F., Gustafsson J.-Å., Balaguer P., Bondesson M. (2019). Differential Activity of BPA, BPAF
and BPC on zebrafish estrogen receptors *in vitro* and *in vivo*. Toxicol. Appl. Pharmacol..

[50] Qiu W., Liu S., Chen H., Luo S., Xiong Y., Wang X., Xu B., Zheng C., Wang K.-J. (2021). The comparative toxicities of BPA,
BPB, BPS, BPF, and BPAF on the reproductive neuroendocrine system
of zebrafish embryos and its mechanisms. J.
Hazard. Mater..

[51] Karim S., Hao R., Pinto C., Gustafsson J.-Å., Grimaldi M., Balaguer P., Bondesson M. (2022). Bisphenol
A analogues induce a feed-forward estrogenic
response in zebrafish. Toxicol. Appl. Pharmacol..

[52] Kubota A., Hirano M., Yoshinouchi Y., Chen X., Nakamura M., Wakayama Y., Lee J. S., Nakata H., Iwata H., Kawai Y. K. (2023). *In Vivo* and *in Silico* Assessments of Estrogenic Potencies
of bisphenol A and its analogs
in zebrafish (*danio rerio*): validity of *in
silico* approaches to predict *in vivo* effects. Comp. Biochem. Physiol., Part C:Toxicol. Pharmacol..

[53] Escher B. I., Henneberger L., König M., Schlichting R., Fischer F. C. (2020). Cytotoxicity burst?
differentiating specific from nonspecific
effects in tox21 in vitro reporter gene assays. Environ. Health Perspect..

[54] Escher B. I., Neale P. A. (2020). Effect-based trigger
values for mixtures of chemicals
in surface water detected with *in vitro* bioassays. Environ. Toxicol. Chem..

[55] Srebny V., Henneberger L., König M., Huchthausen J., Braasch J., Escher B. I. (2025). Beyond
estrogenicity: a comparative
assessment of bisphenol A and its alternatives in in vitro assays
questions safety of replacements. Environ. Sci.
Technol..

[56] Jarque S., Fetter E., Veneman W. J., Spaink H. P., Peravali R., Strähle U., Scholz S. (2018). An automated screening method for
detecting compounds with goitrogenic activity using transgenic zebrafish
embryos. PLoS One.

[57] Nöth J., Busch W., Tal T., Lai C., Ambekar A., Kießling T. R., Scholz S. (2024). Analysis of vascular
disruption in
zebrafish embryos as an endpoint to predict developmental toxicity. Arch. Toxicol..

[58] Sonavane M., Creusot N., Maillot-Maréchal E., Péry A., Brion F., Aït-Aïssa S. (2016). Zebrafish-based
reporter
gene assays reveal different estrogenic activities in river waters
compared to a conventional human-derived assay. Sci. Total Environ..

[59] Grimaldi M., Boulahtouf A., Toporova L., Balaguer P. (2019). Functional profiling
of bisphenols for nuclear receptors. Toxicology.

[60] Pelch K. E., Li Y., Perera L., Thayer K. A., Korach K. S. (2019). Characterization
of estrogenic and androgenic activities for bisphenol A-like chemicals
(BPs): in vitro estrogen and androgen receptors transcriptional activation,
gene regulation, and binding profiles. Toxicol.
Sci..

[61] Delfosse V., Grimaldi M., Pons J.-L., Boulahtouf A., le Maire A., Cavailles V., Labesse G., Bourguet W., Balaguer P. (2012). Structural and mechanistic insights into bisphenols
action provide guidelines for risk assessment and discovery of bisphenol
A substitutes. Proc. Natl. Acad. Sci. U.S.A..

[62] European Chemicals Agency . Key Areas of Regulatory Challenge.; Publications Office: LU. 2023 10.2823/568850.

[63] Opitz R., Maquet E., Huisken J., Antonica F., Trubiroha A., Pottier G., Janssens V., Costagliola S. (2012). Transgenic
zebrafish illuminate the dynamics of thyroid morphogenesis and its
relationship to cardiovascular development. Dev. Biol..

[64] Erradhouani C., Geffroy F., Piccini B., Hinfray N., Chadili E., Balaguer P., Sohm F., Aït-Aïssa S., Coumoul X., Brion F. (2025). A new alternative method using *cyp3a65* expression in transgenic zebrafish embryos to assess
metabolic endocrine-disrupting chemicals in the intestine. Environ. Int..

[65] Billat P.-A., Brochot C., Brion F., Beaudouin R. (2023). A PBPK model
to evaluate zebrafish eleutheroembryos’ actual exposure: bisphenol
A and analogs’ (AF, F, and S) case studies. Environ. Sci. Pollut Res..

[66] Le
Fol V., Brion F., Hillenweck A., Perdu E., Bruel S., Aït-Aïssa S., Cravedi J.-P., Zalko D. (2017). Comparison
of the *in vivo* biotransformation of two emerging
estrogenic contaminants, BP2 and BPS, in zebrafish embryos and adults. Int. J. Mol. Sci..

[67] Loerracher A.-K., Braunbeck T. (2021). Cytochrome
P450-dependent biotransformation capacities
in embryonic, juvenile and adult stages of zebrafish (*Danio
Rerio*)—a state-of-the-art review. Arch. Toxicol..

[68] Verbueken E., Bars C., Ball J. S., Periz-Stanacev J., Marei W. F. A., Tochwin A., Gabriëls I. J., Michiels E. D. G., Stinckens E., Vergauwen L., Knapen D., Van Ginneken C. J., Van Cruchten S. J. (2018). From mRNA
expression of drug disposition genes to in vivo assessment of CYP-mediated
biotransformation during zebrafish embryonic and larval development. Int. J. Mol. Sci..

[69] Cano-Nicolau J., Garoche C., Hinfray N., Pellegrini E., Boujrad N., Pakdel F., Kah O., Brion F. (2016). Several synthetic
progestins disrupt the glial cell specific-brain aromatase expression
in developing zebra fish. Toxicol. Appl. Pharmacol..

[70] Pellegrini E., Diotel N., Vaillant-Capitaine C., Pérez Maria R., Gueguen M.-M., Nasri A., Cano Nicolau J., Kah O. (2016). Steroid modulation of neurogenesis: focus on radial glial cells in
zebrafish. J. Steroid Biochem. Mol. Biol..

[71] Diotel N., Vaillant C., Gabbero C., Mironov S., Fostier A., Gueguen M.-M., Anglade I., Kah O., Pellegrini E. (2013). Effects of
estradiol in adult neurogenesis and brain repair in zebrafish. Horm. Behav..

[72] Vaillant C., Gueguen M.-M., Feat J., Charlier T. D., Coumailleau P., Kah O., Brion F., Pellegrini E. (2020). Neurodevelopmental
effects of natural
and synthetic ligands of estrogen and progesterone receptors in zebrafish
eleutheroembryos. Gen. Comp. Endrocrinol..

[73] Blanc-Legendre M., Sire S., Christophe A., Brion F., Bégout M.-L., Cousin X. (2023). Embryonic Exposures
to Chemicals Acting on Brain Aromatase
Lead to Different locomotor effects in zebrafish larvae. Environ. Toxicol. Pharmacol..

[74] Kinch C. D., Ibhazehiebo K., Jeong J.-H., Habibi H. R., Kurrasch D. M. (2015). Low-dose
exposure to bisphenol A and replacement bisphenol S induces precocious
hypothalamic neurogenesis in embryonic zebrafish. Proc. Natl. Acad. Sci. U.S.A..

[75] Blanc-Legendre M., Guillot L., Chevalier L., Malleret C., Le Menach K., Pardon P., Budzinski H., Brion F., Sire S., Coumailleau P., Charlier T. D., Pellegrini E., Cousin X. (2025). Long-term impact of
embryonic exposure to ethinylestradiol
and clotrimazole on behavior and neuroplasticity in zebrafish (*Danio Rerio*). Environ. Toxicol. Pharmacol..

[76] Malleret C., Blanc-Legendre M., Guillot L., Lautrette-Quinveros H., Pavlidi P., Dalla C., Kokras N., Brion F., Toupin M., Chalmel F., Cousin X., Charlier T. D., Pellegrini E. (2025). Mutation of
brain aromatase impairs behavior and neuroplasticity
in adult zebrafish. J. Neurochem..

[77] Huffman L. S., O’Connell L. A., Hofmann H. A. (2013). Aromatase regulates aggression in
the african cichlid fish *Astatotilapia Burtoni*. Physiol. Behav..

