# Supplementary material for: An Integrated Approach Combining Regulatory Tests in tg(cyp19a1b:GFP) Zebrafish Embryos to Assess Toxicity, Developmental Effects, and Estrogenic Activity of Chemicals: A Case Study with Bisphenol A Substitutes
Source: Environ Sci Technol. 2026 Apr 10;60(16):12046–62. doi: 10.1021/acs.est.5c17875 (PMC13130965; doi:10.1021/acs.est.5c17875)
Supplement: Supplementary file 2 [file es5c17875_si_002.pdf]

An integrated approach combining regulatory tests in *tg(cyp19a1b:GFP)* zebrafish embryos to assess toxicity, developmental effects, and estrogenic activity of chemicals: a case study with bisphenol A substitutes

Florian Geffroy[1,2,#], Armelle Christophe[1,#], Benjamin Piccini[1], Nathalie Hinfray[1], Edith Chadili[1], Emmanuelle Maillot-Marechal[1], Xavier Cousin[3], Mélanie Blanc-Legendre[3], Thierry D. Charlier[4,5,$], Pascal Pandard[1], Selim Aït-Aïssa[1] and François Brion[1]*

[1] Institut National de l'Environnement Industriel et des Risques (INERIS), Parc Technologique ALATA BP2, F-60550 Verneuil-en-Halatte, France

[2] Université Paris Cité, Inserm, HealthFex, F-75006 Paris, France

[3] MARBEC, Université de Montpellier, CNRS, Ifremer, IRD, Inrae, F-34250 Palavas-Les-Flots, France

[4] Université de Rennes, Inserm, EHESP, Irset (Institut de Recherche en Santé, Environnement et Travail), F-35000 Rennes, France

[5] ImPACcell Platform, Biosit, Université de Rennes, F-35000 Rennes, France.

$ Current address: Université de Rennes, CNRS, Normandie Université, EthoS (Éthologie animale et humaine), UMR 6552, F-35000 Rennes, France

* Corresponding author: François Brion, E-mail: francois.brion@ineris.fr; phone +33(0)3 44 55 65 12

# These two authors contributed equally to this work

Tables: 3

Figures: 6

Dataset: 1 (all data generated in this study are compiled in Dataset S1 excel file)

Table S1. Nominal concentrations of bisphenols tested in the refined Fish Embryo Toxicity (FET) assay.

Chemicals	Solvent	Percentage (%)	Concentrations tested (mg/L, nominal)	Concentrations tested (μM, nominal)
TCBPA	DMSO	0.01	0.125, 0.25, 0.5, 1, 2	0.34, 0.68, 1.37, 2.73, 5.46
BPAF	DMSO	0.01	0.25, 0.5, 1, 2, 4	0.74, 1.49, 2.98, 5.95, 11.9
BPS-MPE	DMSO	0.1	0.62, 1.85, 5.56, 16.7, 50	1.82, 5.43, 16.3, 49.0, 147
BPC-Cl	DMSO	0.1	0.99, 2.96, 8.89, 26.7, 80	3.52, 10.5, 31.6, 94.9, 285
BPC	DMSO	0.1	0.32, 0.95, 2.84, 8.53, 25.6	1.25, 3.71, 11.1, 33.3, 99.8
BPB	None	-	0.625, 1.25, 2.5, 5, 10	2.58, 5.16, 10.3, 20.7, 41.3
BPA	DMSO	0.01	1.25, 2.5, 5, 10, 20	5.47, 10.95, 21.9, 43.8, 87.6
4,4'ODP	DMSO	0.01	0.99, 2.96, 8.89, 26.7, 80	4.90, 14.6, 43.9, 132, 395
BPS-MAE	DMSO	0.1	1.23, 3.7, 11.1, 33.3, 100	4.24, 12.75, 38.25, 115, 344
BPF	DMSO	0.5	3.75, 7.5, 15, 30, 60	18.7, 37.5, 74.9, 150, 300
BPS	DMSO	0.1	25, 50, 100, 200, 400	100, 200, 400, 800, 1600

Table S2. Nominal concentrations of bisphenols tested in the EASZY assay.

Chemicals	Solvent	Percentage (%)	Concentrations tested (mg/L, nominal)	Concentrations tested (μM, nominal)
TCBPA	DMSO	0.01	0.0156, 0.0312, 0.0625, 0.125, 0.25	0.043, 0.085, 0.171, 0.342, 0.684
BPAF	DMSO	0.01	0.021, 0.042, 0.085, 0.17, 0.34	0.062, 0.125, 0.253, 0.506, 1.012
BPS-MPE	DMSO	0.01	0.125, 0.25, 0.5, 1, 2	0.367, 0.734, 1.47, 2.94, 5.87
BPC-Cl	DMSO	0.01	0.00003, 0.0003, 0.003, 0.03, 0.3	0.00011, 0.00107, 0.0107, 0.107, 1.07
BPC	DMSO	0.01	0.0625, 0.125, 0.25, 0.5, 1	0.244, 0.488, 0.977, 1.955, 3.91
BPB	DMSO	0.01	0.078, 0.156, 0.312, 0.625, 1.25	0.322, 0.644, 1.29, 2.58, 5.15
BPA	DMSO	0.01	0.312, 0.625, 1.25, 2.5, 5	1.366, 2.73, 5.46, 10.93, 21.86
4,4'ODP	DMSO	0.01	0.0123, 0.037, 0.11, 0.33, 1	0.061, 0.183, 0.544, 1.63, 4.94
BPS-MAE	DMSO	0.01	0.625, 1.25, 2.5, 5, 10	2.15, 4.31, 8.62, 17.24, 34.48
BPF	DMSO	0.01	0.012, 0.037, 0.11, 0.33, 1	0.060, 0.185, 0.55, 1.65, 5.0
BPS	DMSO	0.1	25, 50, 100, 200, 400	100, 200, 400, 800, 1600

Figure S1. Concentration-response curves of bisphenols in the refined Fish Embryo Toxicity (FET) assay, modeled using the Hill equation to derive the LC_{50}(96h) values (expressed in mg/L, nominal concentrations).

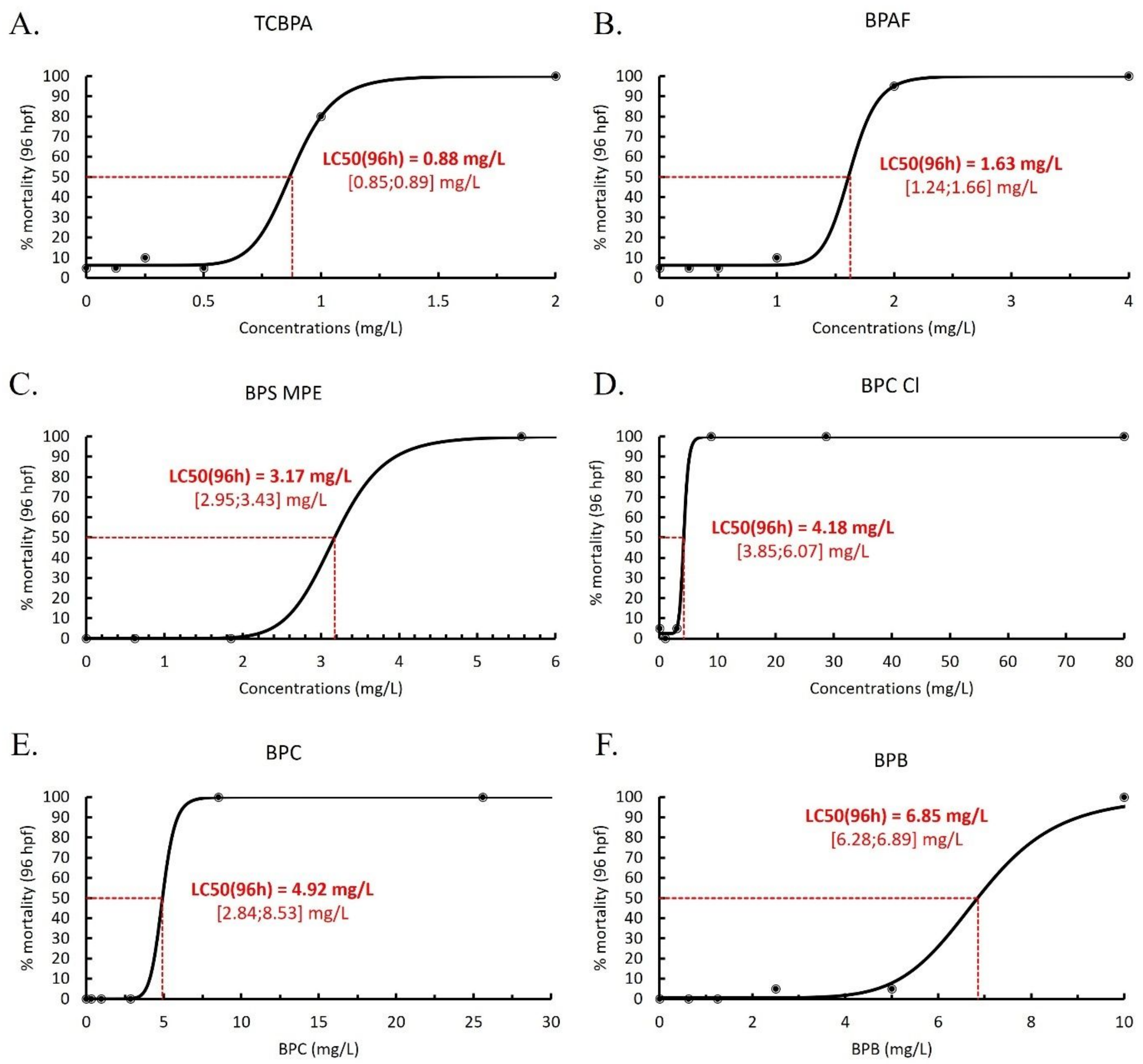

Figure S1. Continued

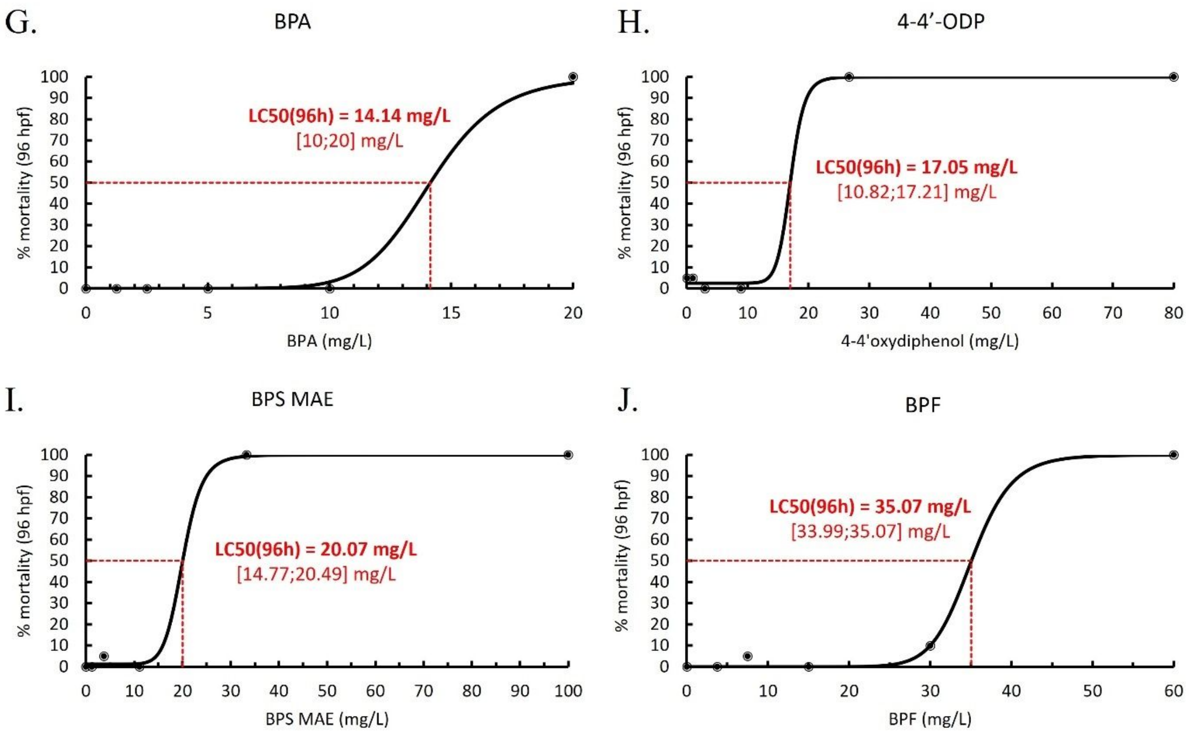

Figure S2. Hatching rates across time for zebrafish embryos exposed to bisphenols at several concentrations (mg/L, nominal).

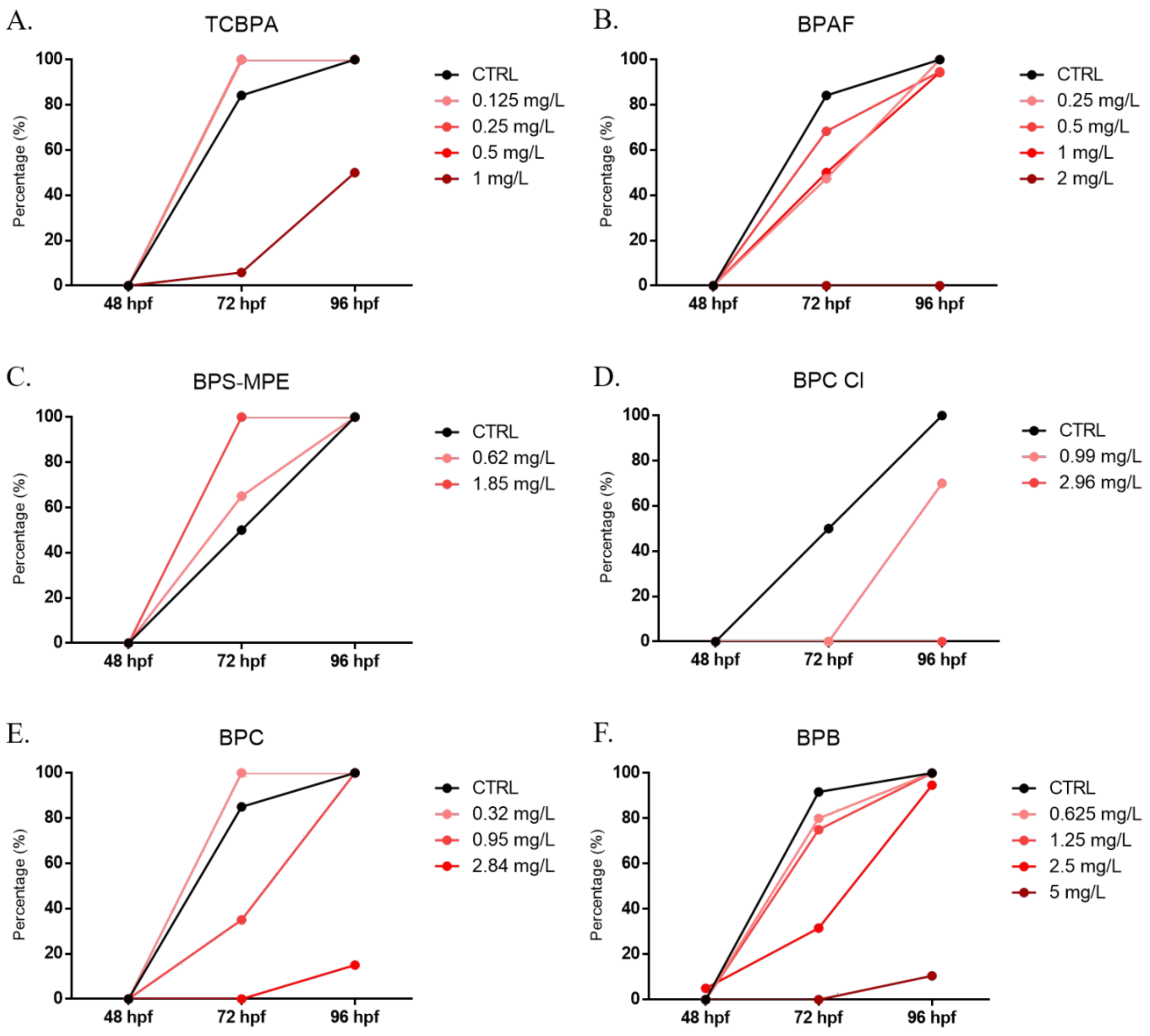

Figure S2. Continued

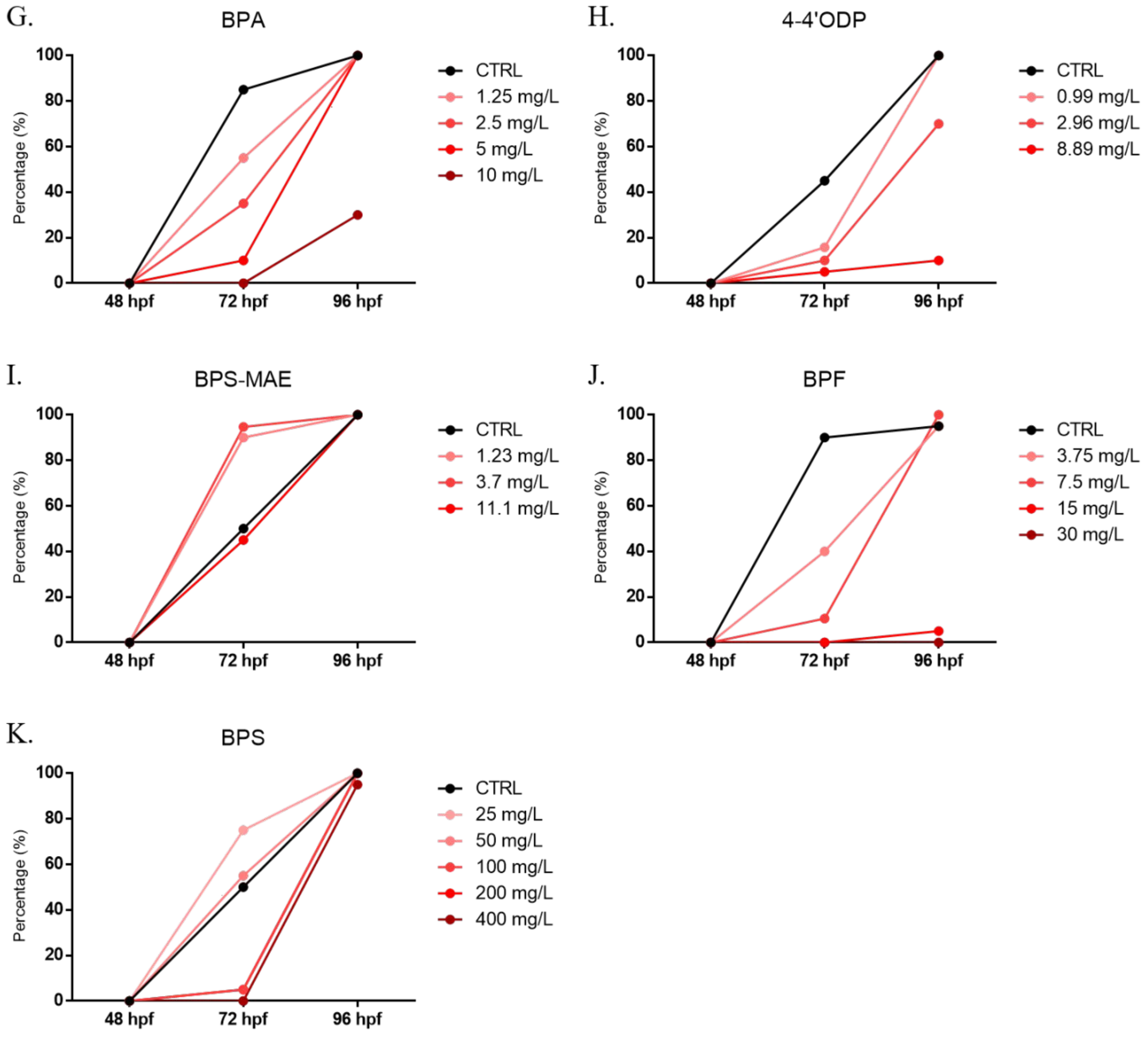

Figure S3. Representative phenotypic malformations observed in zebrafish embryos during the refined Fish Embryo Toxicity (FET) assay after exposure to bisphenols. For each image, the nominal concentration and time of exposure are indicated. Symbols denote the following malformations: deformity of yolk (*), edema (#), no or less pigmentation ($), head malformations (°) and tail/spinal malformations (¤). Images were photographed using a X10 objective.

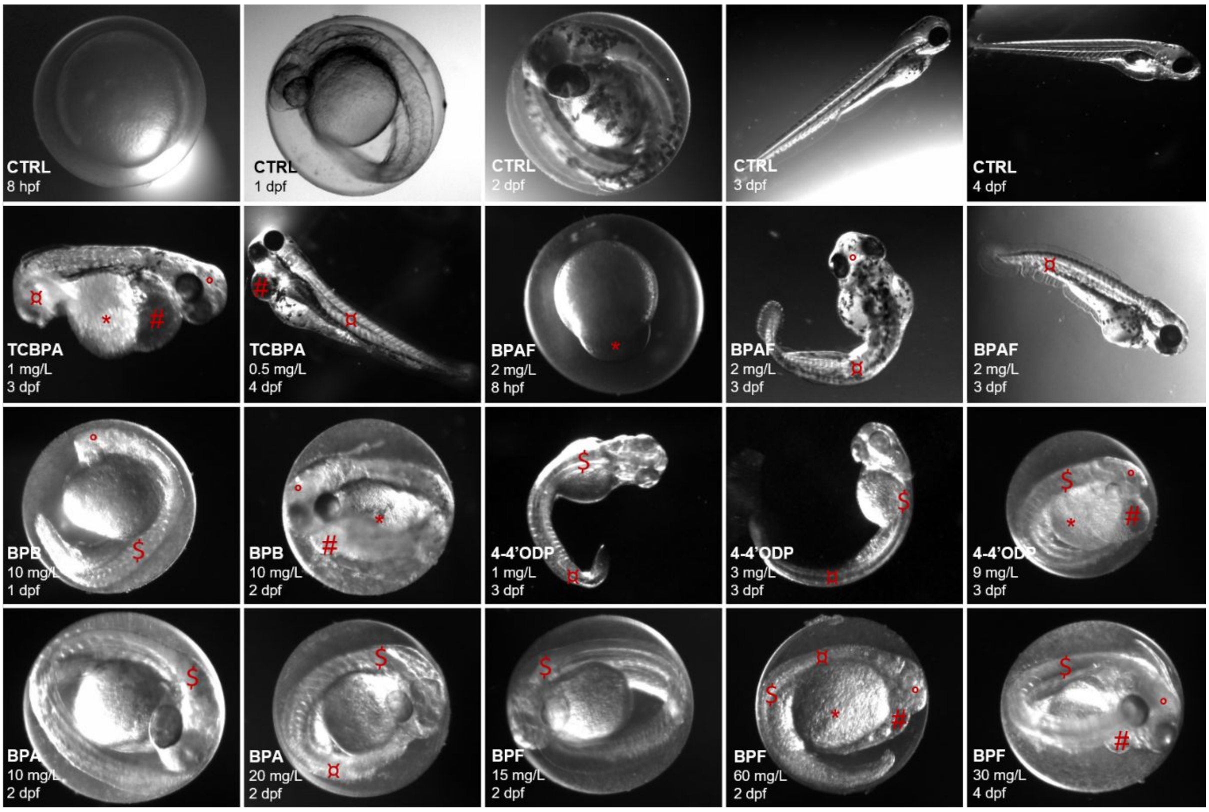

Table S3. Summary of toxic responses and malformations induced by bisphenols in fish: comparison between the present study and literature data.

	Study		Literature			
Chemicals	LC50(96h) [mg/L, nominal]	Malformations	Observations	Species	Test	References
TCBPA	0.88	Deformity of yolk, Spontaneous movement, Edema, Blood tail circulation, Heart rate disruption, Pigmentation, Head, tail/spinal malformations, delayed hatching	Concentrations above 1 μM cause lethal effects, Delayed hatching	Zebrafish Wildtype TAB and Tg(hPPARγ-eGFP)	3-6 dpf exposure	Riu et al. (2014)
			LC50(144h) = 0.75 mg/L; LOAELs =1mg/L; Edema, Hemorrhage, Inhibition of hatching	Zebrafish embryos	150h exposure	Song et al. (2014)
BPAF	1.63	Deformity of yolk, Spontaneous movement, Heart rate disruption, Head, tail/spinal malformations, Hemorrhage	No effect on survival and hatchability, Edema for 0.372 μM BPAF at 96 hpf; BPAF induced p450 aromatase, VTG and E2 at 7d	Zebrafish embryos	7-days exposure	Chen et al. (2018)
			Malformations: Edema, Otic vesicle deformities, Delayed hatching	Zebrafish	Various (review on bisphenols)	Pelch et al. (2019)
			BPAF (0, 0.2, 0.6, 1.8, 5.2, 15.3, or 45.0 μM); NOEC = 1.8 μM; Developmental toxicity: BPAF > BPB > BPF = BPA > BPS	Zebrafish Wildtype (AB/TL)	1-6 dpf static + 6-9 dpf semistatic	Catron et al. (2019)
			LC50(48h) = 4.2 (3.6-4.7) mg/L; Pigmentation : EC50(48h) = 3.3 (2.4-4.5) mg/L; Missing blood flow : EC50(48h) = 3.0 (0.67-13.6) mg/L; Edema	Zebrafish embryos	4-days exposure	Tišler et al. (2016)

	: EC50(48h) = 2.8 (1.4-5.4) mg/L; Hatching inhibition: EC50(72h) = 2.2 (1.8-2.8) mg/L			
BPAF continued	LC50(96h) = 1.6 (0.09) mg/L; Hatching success: EC50(72h) = 0.92 (0.06) mg/L, Delayed hatching; Edema	Zebrafish embryos	4-days exposure	Moreman et al. (2017)
	LC50(96h) = 1.95 (1.72-2.23) mg/L; Delayed hatching, Hearte rate disruption, Edema, tail/spinal malformations	Zebrafish embryos	4-days exposure	Mu et al. (2018)
	LC50(144h) = 1.75 mg/L; LOAELs = 1 mg/L; Edema, Delayed hatching	Zebrafish embryos	150h exposure	Song et al. (2014)
	LC50(48h) = 3.89 mg/L, LC50(72h) = 3.49 mg/L, LC50(96h) = 2.04 mg/L	Zebrafish embryos		Ren et al. (2017)
	Body length: NOEC = 0.5 mg/L, LOEC = 1 mg/L	Zebrafish embryos		
	LC50(24h) = 3.15 mg/L, LC50(48h) = 2.64 mg/L, LC50(72h) = 2.47 mg/L, LC50(96h) = 2.47 mg/L	Adult Zebrafish		
	Reduced body length, decreased movement distance at 120 hpf, increased number of GnRH3 neurons, increased expression of reproductive neuroendocrine-related genes and hormones: LOEC = 100 μg/L, NOEC = 1 μg/L	Zebrafish embryos	120h-exposure	Qiu et al. (2021)
	LC50(96h) = 8.24 μM, Exposure to BPAF in μM (between 2 to 5μM) reduced heart rate at 48hpf	Zebrafish embryos (Wildtype AB)	96h-exposure	Arrokhman et al. (2023)

BPS MPE	3.17	Deformity of yolk, Edema, Heart rate disruption, Pigmentation, tail/spinal malformations, Hemorrhage	-	-	-	-
BPC-Cl	4.18	Deformity of yolk, Spontaneous movement, Edema, Blood tail circulation, Heart rate disruption, Pigmentation, tail/spinal malformations, Hemorrhage	-	-	-	-
BPC	4.92	Deformity of yolk, Edema, Blood tail circulation, Heart rate disruption, tail/spinal malformations, Hemorrhage	-	-	-	-
BPB	6.85	Deformity of yolk, Spontaneous movement, Edema, Heart rate disruption, Head malformations, Hemorrhage	Malformations: Edema, Otic vesicle deformities	Zebrafish	Various (review on bisphenols)	Pelch et al. (2019)
			BPB (0, 0.6, 1.7, 5.1, 15.0, or 44.0 mM); NOEC = 5.1 µM; Developmental toxicity: BPAF > BPB > BPF = BPA > BPS	Zebrafish Wildtype (AB/TL)	1-6 dpf static + 6-9 dpf semistatic	Catron et al. (2018)
			Yeast two-hybrid assay, estrogenicity assessment: BPB ≥ BPA, BPF > BPS; decrease of activity probably due to higher acute toxicity	Daphnia magna	acute toxicity assay	Chen et al. (2002)

BPB continued			LC50(24h) = 7.18 mg/L, LC50(48h) = 6.54 mg/L, LC50(72h) = 4.53 mg/L, LC50(96h) = 3.88 mg/L	Zebrafish embryos		Ren et al. (2017)
			Body length: NOEC = 1 mg/L, LOEC = 2 mg/L	Zebrafish embryos		
			LC50(24h) = 5.07 mg/L, LC50(48h) = 4.64 mg/L, LC50(72h) = 4.15 mg/L, LC50(96h) = 4.15 mg/L	Adult Zebrafish		
			Reduced body length, decreased movement distance at 120 hpf, increased number of GnRH3 neurons, increased expression of reproductive neuroendocrine-related genes and hormones: LOEC = 100 µg/L, NOEC = 1 µg/L	Zebrafish embryos	120h-exposure	Qiu et al. (2021)
			LC50(96h) = 28 µM, Exposure to BPB in µM (between 6 to 15µM) reduced heart rate at 48hpf	Zebrafish embryos (Wildtype AB)	96h-exposure	Arrokhman et al. (2023)
BPA	14.14	Deformity of yolk, Growth retardation, Spontaneous movement, Edema, Heart rate disruption, Pigmentation, tail/spinal malformations, Hemorrhage	100 µM (23 mg/L): 100% mortality at 72h, developmental and comportemental effects; 100 nM (0,023 mg/L): no effect of BPA	Zebrafish embryos	5-days exposure	Björnsdotter et al. (2017)
			LC25 = 2.8x10-6 M, NOAEL ≥ 10-6 M, non-teratogen	Zebrafish embryos	120h exposure	Brannen et al. (2010)
			EC50 = 1226 µg/L [1169;1310] = 1370 nM, LOEC = 1000 µg/L = 4380.32 nM, No developmental toxicity	Zebrafish embryos ERE-GFP-Casper	120h exposure	Green et al. (2016)

BPA continued	LC50(24h) = 16.75 mg/L, NOEC = 2 mg/L, 100% mortality at 25 mg/L; Edema, Blood flow, Delayed hatching	Zebrafish embryos	72h exposure	Duan et al. (2008)
	Exposure to 20 mg/L of BPA; 24h: Deformity of yolk, Edema, Growth retardation, Head, tail/spinal malformations; 48h: 65% mortality, Heart rate disruption, Head malformations, Pigmentation, Edema	Zebrafish Wildtype (AB/TL)	48h exposure	Makarova et al. (2016)
	LC50(96h) = 12.8 mg/L	Zebrafish	4-days exposure	Corrales et al. (2017)
	LC50(96h) = 4.2 mg/L	Fathead minnow	4-days exposure	Corrales et al. (2017)
	LC50 = 17.5 ± 0.37 µM (= 3.995 ± 0.08 mg/L); 5-15 µM induced 4-8% mortality; Edema, Hemorrhage, tail/spinal malformations, Delayed hatching	Zebrafish embryos	5-days exposure	McCormick et al. (2010)
	LC50(5dpf) = 5 (±0.89) mg/L, LC50(28dpf) = 1.8 (±0.23) mg/L, LOAEL(5dpf) = 1 mg/L, LOAEL(28dpf) = nd; Edema, Hemorrhage, Curved tails, Delayed hatching	Zebrafish embryos	7-days exposure	McCormick et al. (2011)
	1000 µg/L induce no mortality at 120 hpf, increase hatching at 48 and 54 hpf, Body length reduced in concentration-dependent manner	Zebrafish embryos	120h exposure	Qiu et al. (2018)

	BPA (1, 5, and 15 µM) have no effect on the development between 8 and 96 hpf; BPA alters motor behavior of larvae between 18 and 120 hpf	Zebrafish embryos (Wildtype AB)	120h exposure	Wang et al. (2013)
BPA continued	Exposure to 10-100 µM of BPA; No mortality until 70 µM, no malformations below 30 µM; 30-70 µM : Edema, Craniofacial abnormalities, Delayed hatching; Transient exposure to 0.01 and 0.1 µM BPA resulted in significant larval hyperactivity (no effect for 1 and 10 µM).	Zebrafish embryos	120h exposure	Saili et al. (2012)
	Transient exposure to 0.1 and 1 µM BPA results in learning deficits in adults (more trials to learn in T-maze)	Zebrafish adults	120h exposure	Saili et al. (2012)
	Exposure to 0.1, 1, 10, 100, 1000 µg/L with renewal every 12h and final DMSO concentration at 0.005% v/v; No effect on survival and time of hatching; Malformations: Spinal curvature, Edema	Zebrafish embryos (Wildtype AB)	4-168h exposure	Wu et al. (2011)
	BPA induced GFP in liver and heart; BPA has effects on the heart, in its structure but not in its cardiovascular function	Zebrafish embryos/larvae TG(ERE:GFP)	5-days exposure or 6-15 dpf exposure	Brown et al. (2019)
	Malformations: Edema, Otic vesicle deformities	Zebrafish	Various (review on bisphenols)	Pelch et al. (2019)

	BPA (0, 0.2, 0.6, 1.7, 2.9, 5.7, 11.5, 23.0, or 45.0 mM); NOEC = 11.5 µM; Developmental toxicity: BPAF > BPB > BPF = BPA > BPS	Zebrafish Wildtype (AB/TL)	1-6 dpf static exposure + 6-9 dpf semistatic exposure	Catron et al. (2018)
BPA continued	Yeast two-hybrid assay, estrogenicity assessement: BPB ≥ BPA, BPF > BPS	Daphnia magna	acute toxicity assay	Chen et al. (2002)
	Renewal of test media every 12h, higher hatching rates at 48 and 55 hpf for 1 and 10 µg/L, no effect on survival until 1000 µg/L	Zebrafish GnRH3-EMD transgenic	120h exposure	Qiu et al. (2016)
	LC50(48h) = 15.9 (13.8-18.3) mg/L; Pigmentation : EC50(48h) = 3.6 (2.5-5.3) mg/L; Missing blood flow : EC50(48h) = 12.3 (10.3-14.6) mg/L; Edema : EC50(48h) = 13.9 (8.5-22.8) mg/L; Hatching inhibition: EC50(72h) = 4.0 (3.1-5.2) mg/L	Zebrafish embryos	4-days exposure	Tišler et al. (2016)
	LC50(96h) = 10.4 (9.44-11.58) mg/L; Edema	Zebrafish embryos	4-days exposure	Mu et al. (2018)
	LC50(144h) = 7.5 mg/L; LOAELs = 5 mg/L	Zebrafish embryos	150h exposure	Song et al. (2014)
	LC50(96h) = 12 (0.22) mg/L; Hatching success: EC50(72h) = 5;7 (0.33) mg/L, Delayed hatching; Edema, Craniofacial abnormality, Hemorrhage	Zebrafish embryos	4-days exposure	Moreman et al. (2017)
	LC50(96h) = 9.82 mg/L, LC10(96h) = 6.62 mg/L	Zebrafish embryos	4-days exposure	Blanc et al. (2019)

BPA continued	LC50(96h) = 35 µM = 8.041 mg/L [7.846;8.24] mg/L, delayed hatching: EC50(96h) = 5.25 mg/L [2.982;5.819] mg/L	Zebrafish embryos	4-days exposure	Chow et al. (2013)
	Mortality at 219 µM (50 mg/L), delayed hatching from 35 µM (8.041 mg/L), inflated swim bladder from 17.5 µM (4 mg/L), scoliosis (16% at 8.041 mg/L); Reduced body length, deformity of yolk, head and spinal malformations, pigmentation (malformations occurring at low concentrations)	Zebrafish embryos	2-5 dpf exposure	Martínez et al. (2019)
	Body length: NOEC = 2 mg/L, LOEC = 4 mg/L	Zebrafish embryos		Ren et al. (2017)
	LC50(24h) = 9.51 mg/L, LC50(48h) = 9.31 mg/L, LC50(72h) = 8.09 mg/L, LC50(96h) = 8.09 mg/L	Adult Zebrafish		
	Malformations observed: axial malformation, pericardial edema, yolk sac edema (72 hpf); exposure to 0.001 mg/L of BPA	Zebrafish embryos	72h-exposure	Üstündağ et al. (2017)
	Accelerated hatching, reduced body length, decreased movement distance at 120 hpf, increased number of GnRH3 neurons, increased expression of reproductive neuroendocrine-related genes and hormones: LOEC = 100 µg/L, NOEC = 1 µg/L	Zebrafish embryos	120h-exposure	Qiu et al. (2021)

BPA continued			Exposure to 1 and 100 µg/L of BPA increased sinus venosus-bulbus arteriosus (SV-BA) distance; increased heart rate at 36 hpf but decreased it at 72 and 120 hpf	Zebrafish embryos (Wildtype AB)	96h-exposure	Qin et al. (2023)
			LC50(96h) = 57.1 µM,<br>Exposure to BPA in µM (between 5 to 35µM) reduced heart rate at 48hpf	Zebrafish embryos (Wildtype AB)	96h-exposure	Arrokhman et al. (2023)
4-4'-ODP	17.05	Deformity of yolk, Growth retardation, Edema, Blood tail circulation, Heart rate disruption, Pigmentation, Head, tail/spinal malformations, Hemorrhage	-	-	-	-
BPS MAE	20.07	Deformity of yolk, Spontaneous movement, Edema, Blood tail circulation, Hearte rate disruption, Pigmentation, tail/spinal malformations	-	-	-	-
BPF	35.07	Deformity of yolk, Spontaneous movement, Edema, Blood tail circulation,	1000 µg/L: no mortality at 120 hpf, increase hatching at 48 and 54 hpf; body length reduced in concentration-dependent manner	Zebrafish embryos	120h-exposure	Qiu et al. (2018)

BPF continued	Heart rate disruption, Pigmentation, Head, tail/spinal malformations, Hemorrhage	LC50(96h) = 10.030 mg/L; Delayed hatching, Edema, Tail/spinal malformations, Hemorrhage, Coagulation	Zebrafish embryos (Wildtype AB)	120h-exposure	Yang et al. (2017)
		BPF (0, 0.2, 0.6, 1.8, 5.2, 15.3, 45.0 mM); NOEC = 15.3 µM; Developmental toxicity: BPAF > BPB > BPF = BPA > BPS	Zebrafish Wildtype (AB/TL)	1-6 dpf + 6-9 dpf	Catron et al. (2018)
		Only 20% mortality at 20 mg/L; Pigmentation: EC50(48h) = 1.1 (0.92-1.3) mg/L; Edema: EC50(48h) = 10.7 (9.4-12.2) mg/L; Hatching inhibition: EC50(72h) = 6.8 (5.8-8.5) mg/L	Zebrafish embryos	4-days exposure	Tišler et al. (2016)
		LC50(96h) = 32 (0.55) mg/L; Hatching success: EC50(72h) = 14 (0.41) mg/L, Delayed hatching; Edema, Craniofacial abnormality, Tail development, Hemorrhage, Deformity of yolk	Zebrafish embryos	4-days exposure	Moreman et al. (2017)
		LC50(96h) = 19.6 (18.47-20.67) mg/L; Delayed hatching, Hearte rate disruption, Edema, tail/spinal malformations, Pigmentation	Zebrafish embryos	4-days exposure	Mu et al. (2018)
		LC50(24h) = 9.13 mg/L, LC50(48h) = 8.93 mg/L, LC50(72h) = 8.56 mg/L, LC50(96h) = 7.40 mg/L	Zebrafish embryos		Ren et al. (2017)
		Body length: NOEC = 6 mg/L, LOEC = 8 mg/L	Zebrafish embryos		

BPF continued			LC50(24h) = 10.10 mg/L, LC50(48h) = 9.86 mg/L, LC50(72h) = 9.51 mg/L, LC50(96h) = 9.51 mg/L<br>LC50(96h) = 7.40 mg/L	Adult Zebrafish		Gu et al. (2020)
			Accelerated hatching, reduced body length, increased number of GnRH3 neurons, increased expression of reproductive neuroendocrine-related genes and hormones: LOEC = 100 µg/L, NOEC = 1 µg/L	Zebrafish embryos	120h-exposure	Qiu et al. (2021)
			Exposure to 1 and 100 µg/L of BPF decreased sinus venosus-bulbus arteriosus (SV-BA) distance; increased heart rate at 36, 72 and 120 hpf<br>Exposure to 1 µg/L increased stroke volume	Zebrafish embryos (Wildtype AB)	96h-exposure	Qin et al. (2023)
			LC50(96h) = 119.7 µM,<br>Exposure to BPF in µM (between 15 to 60µM) reduced heart rate at 48hpf	Zebrafish embryos (Wildtype AB)	96h-exposure	Arrokhman et al. (2023)
BPS	CNC	Deformity of yolk	1000 µg/L: no mortality at 120 hpf, increase hatching at 48 and 54 hpf, Body length reduced in concentration-dependent manner	Zebrafish embryo	120h exposure	Qiu et al. (2018)
			Malformations: Edema, Otic vesicle deformities, Delayed hatching	Zebrafish	Various (review on bisphenols)	Pelch et al. (2019)

BPS continued	BPS (0, 0.2, 0.6, 1.8, 5.2, 15.3, 45.0 mM); NOEC = 45 µM, no toxicity for BPS; Developmental toxicity: BPAF > BPB > BPF = BPA > BPS	Zebrafish Wildtype (AB/TL)	1-6 dpf static exposure + 6-9 dpf semi-static exposure	Catron et al. (2018)
	Yeast two-hybrid assay, estrogenicity assessement: BPB ≥ BPA, BPF > BPS	Daphnia magna	acute toxicity assay	Chen et al. (2002)
	BPS (0, 1, 3, 10, 30 µg/L), DMSO 0.01% (v/v); No effect on survival or growth at 168 hpf, no significative malformations, Delayed hatching	Zebrafish embryos (Wildtype AB)	168h exposure	Zhang et al. (2017)
	Renewal of test media every 12h, no mortality for 100 µg/L	Zebrafish GnRH3-EMD transgenic	120h exposure	Qiu et al. (2016)
	LC50(96h) = 199 (7.6) mg/L; Hatching success: EC50(72h) = 155 (15) mg/L, Delayed hatching; Edema, Craniofacial abnormality, Tail development	Zebrafish embryos	4-days exposure	Moreman et al. (2017)
	No mortality and no malformations until 50 mg/L	Zebrafish embryos	4-days exposure	Mu et al. (2018)
	LC50(96h) > 400 µM (> 100 mg/L), LC10(96h) > 400 µM (> 100 mg/L)	Zebrafish embryos	4-days exposure	Blanc et al. (2019)
	No mortality, LC50(96h) > 30 µM (> 7.5 mg/L)	Zebrafish embryos	4-days exposure	(Le Fol et al. 2017)
	LC50(24h) = 361 mg/L, LC50(48h) = 346 mg/L, LC50(72h) = 331 mg/L, LC50(96h) = 323 mg/L	Zebrafish embryos		Ren et al. (2017)

BPS continued	Heart rate: EC50(78h) = 318 mg/L; Hatching rate: EC50(120h) = 200 mg/L; Body length: NOEC = 300 mg/L, LOEC = 350 mg/L	Zebrafish embryos		
	LC50(24h) = 343 mg/L, LC50(48h) = 343 mg/L, LC50(72h) = 343 mg/L, LC50(96h) = 343 mg/L	Adult Zebrafish		
	Accelerated hatching, reduced body length, increased number of GnRH3 neurons, increased expression of reproductive neuroendocrine-related genes and hormones: LOEC = 100 μg/L, NOEC = 1 μg/L	Zebrafish embryos	120h-exposure	Qiu et al. (2021)
	Exposure to 1 and 100 μg/L of BPS decreased sinus venosus-bulbus arteriosus (SV-BA) distance; increased heart rate at 36 and 72 hpf<br>Exposure to 1 μg/L increased stroke volume and heart rate at 120 hpf	Zebrafish embryos (Wildtype AB)	96h-exposure	Qin et al. (2023)

Figure S4. GFP fold induction in *tg(cyp19a1b:GFP)* zebrafish embryos exposed to BPS in the refined FET assay, with Hill model fits used to derive relative EC10, EC20, and EC50 values (mg/L, nominal concentrations).

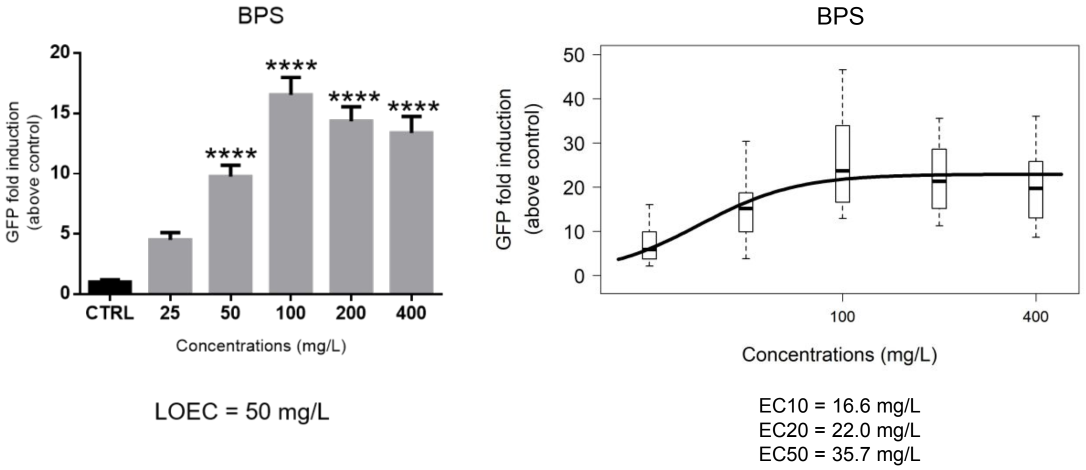

Figure S5. GFP fold induction in the EASZY assay: concentration-response modeling of bisphenols active on brain aromatase, expressed as percentage of the EE2 response, with relative EC_{10}, EC_{20}, and EC_{50} values (mg/L, nominal concentrations)

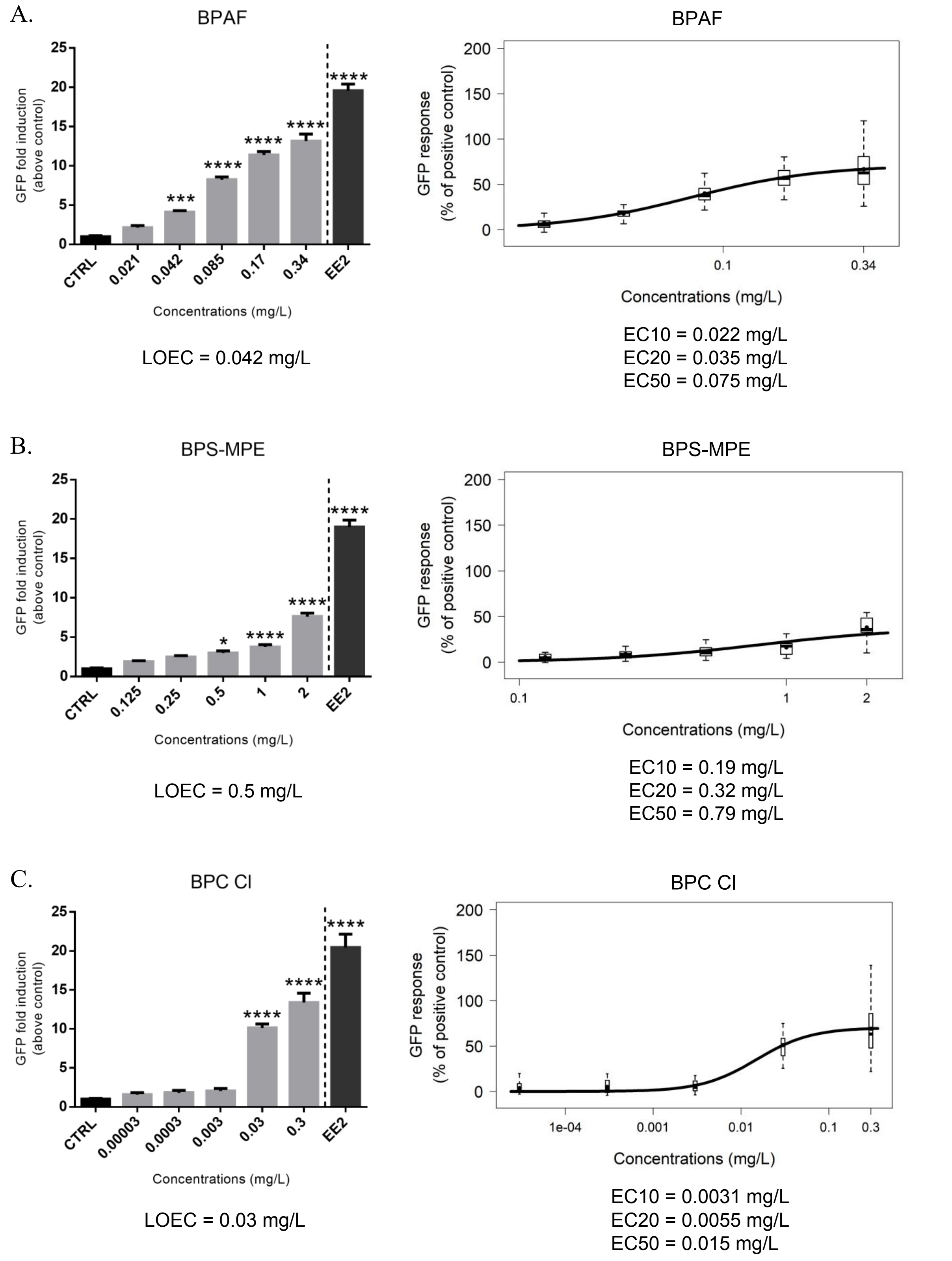

Figure S5. Continued

D.

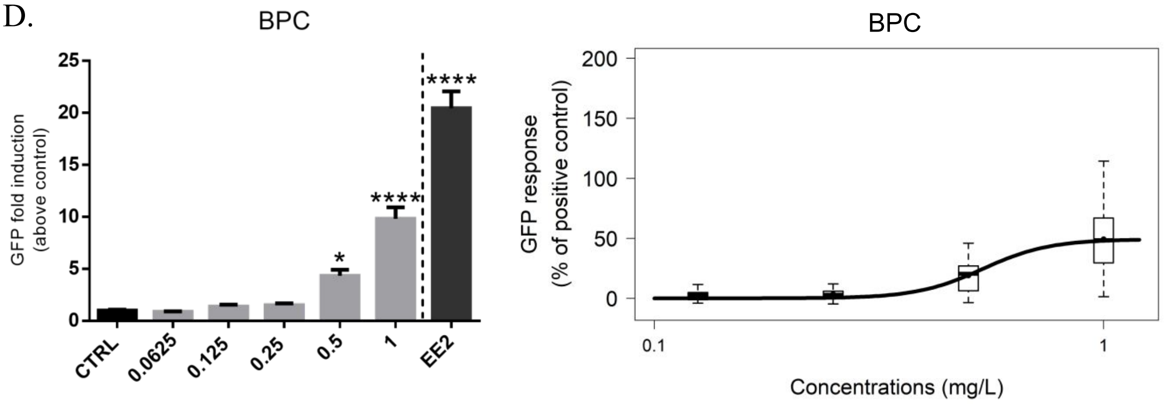

LOEC = 0.5 mg/L

EC10 = 0.37 mg/L
EC20 = 0.425 mg/L
EC50 = 0.54 mg/L

E.

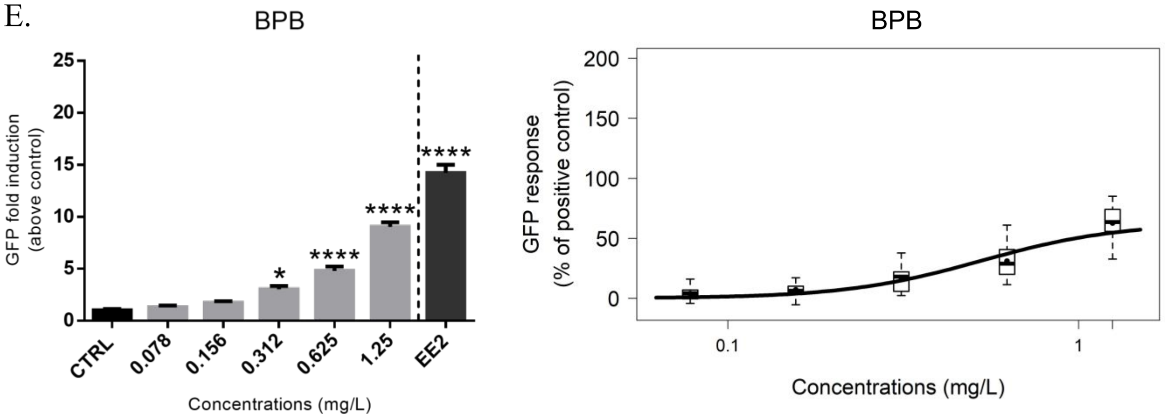

LOEC = 0.312 mg/L

EC10 = 0.20 mg/L
EC20 = 0.28 mg/L
EC50 = 0.53 mg/L

F.

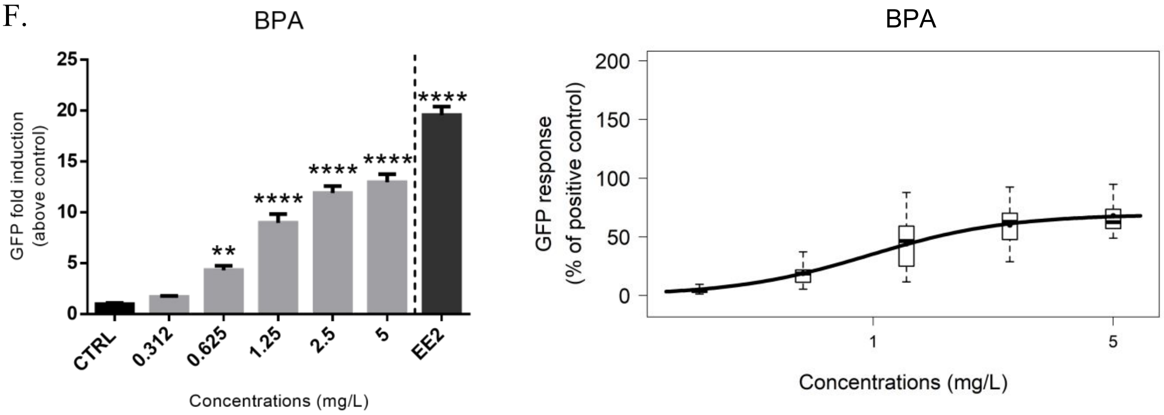

LOEC = 0.625 mg/L

EC10 = 0.36 mg/L
EC20 = 0.52 mg/L
EC50 = 0.98 mg/L

Figure S5. Continued

G.

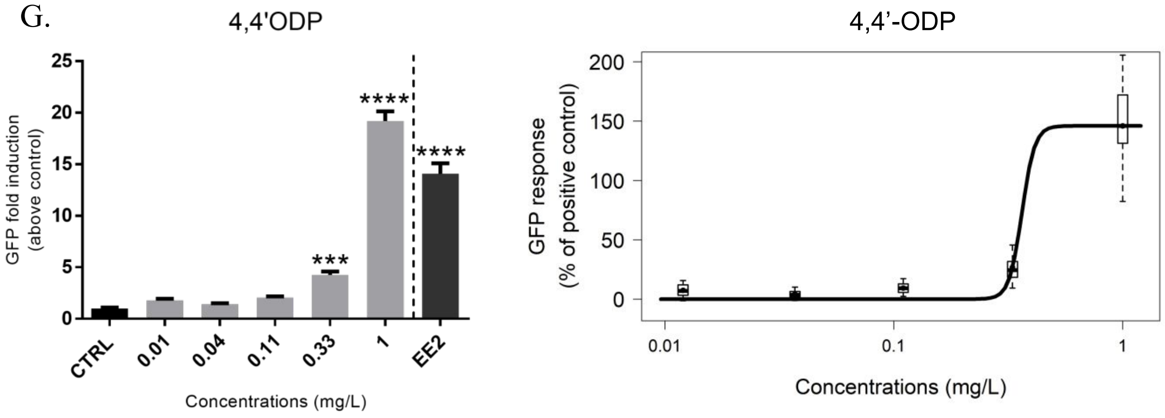

LOEC = 0.33 mg/L

EC10 = 0.315 mg/L
EC20 = 0.33 mg/L
EC50 = 0.36 mg/L

H.

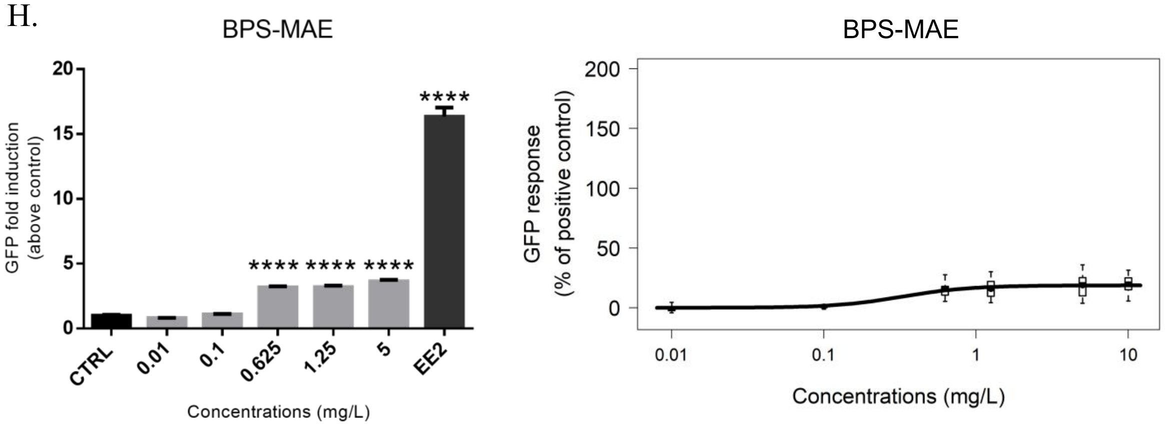

LOEC = 0.625 mg/L

EC10 = 0.105 mg/L
EC20 = 0.16 mg/L
EC50 = 0.33 mg/L

I.

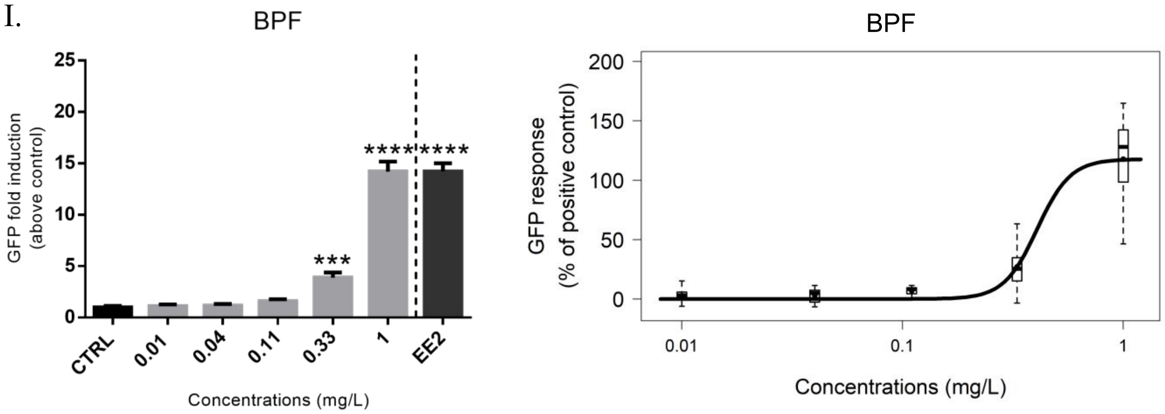

LOEC = 0.33 mg/L

EC10 = 0.28 mg/L
EC20 = 0.32 mg/L
EC50 = 0.41 mg/L

Figure S5. Continued

J.

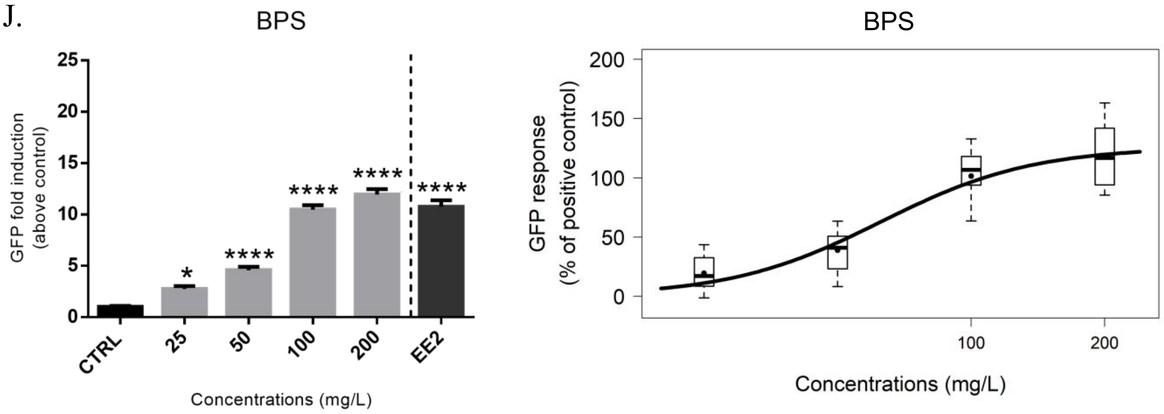

LOEC = 25 mg/L

EC10 = 26.4 mg/L
EC20 = 36.3 mg/L
EC50 = 62.7 mg/L

Figure S6. GFP induction in *tg(cyp19a1b:GFP)* zebrafish embryos co-exposed to bisphenols and the estrogen receptor antagonist ICI 182,780.

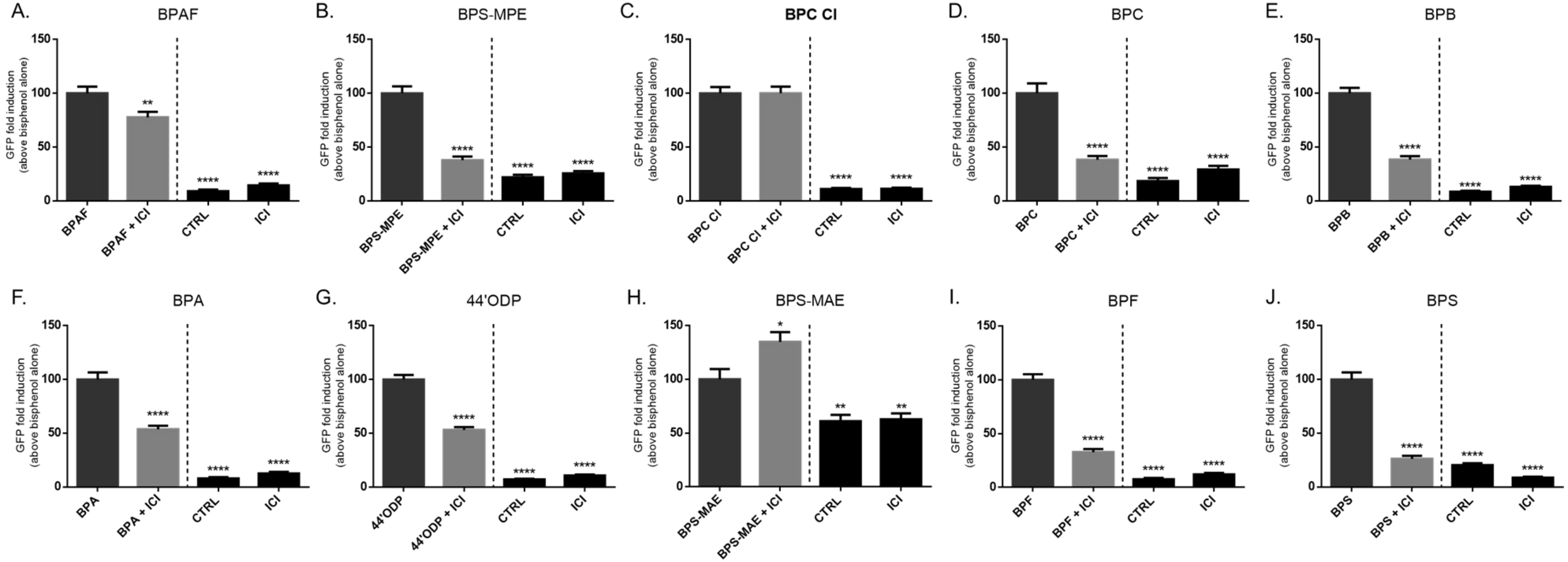

BPAF (0.34 mg/L; 1 µM), BPS-MPE (2 mg/L; 5.9 µM), BPC (1 mg/L; 3.9 µM), BPB (1.25 mg/L; 5.16 µM), BPA (5 mg/L; 22 µM), 4,4'-ODP (1 mg/L; 5 µM), BPF (1 mg/L; 5 µM): 96h co-exposure with bisphenol and ICI 182,780 (1µM).

BPC-Cl (0.3 mg/L; 1.1 µM), BPS-MAE (0.625 mg/L; 2.2 µM), BPS (100 mg/L; 400 µM): 48h pre-exposure with ICI 182,780 followed by 48h co-exposure with bisphenol and ICI 182,780 until 96h. This doesn't work for BPC-Cl and BPS-MAE even with the processing treatment.

GFP induction by bisphenols was normalized to 100%, and the decrease in GFP expression by co-exposure with ICI is expressed as fold reduction.

Figure S7. Induction of luciferase activity in the ZELH-zfERβ2 cell line by bisphenols. (A) 17β-estradiol. (B-J) Active bisphenols. (K-L) Non active bisphenols. Data from 3 independent experiments were pooled to generate the concentration-response curves. All concentrations are nominal.

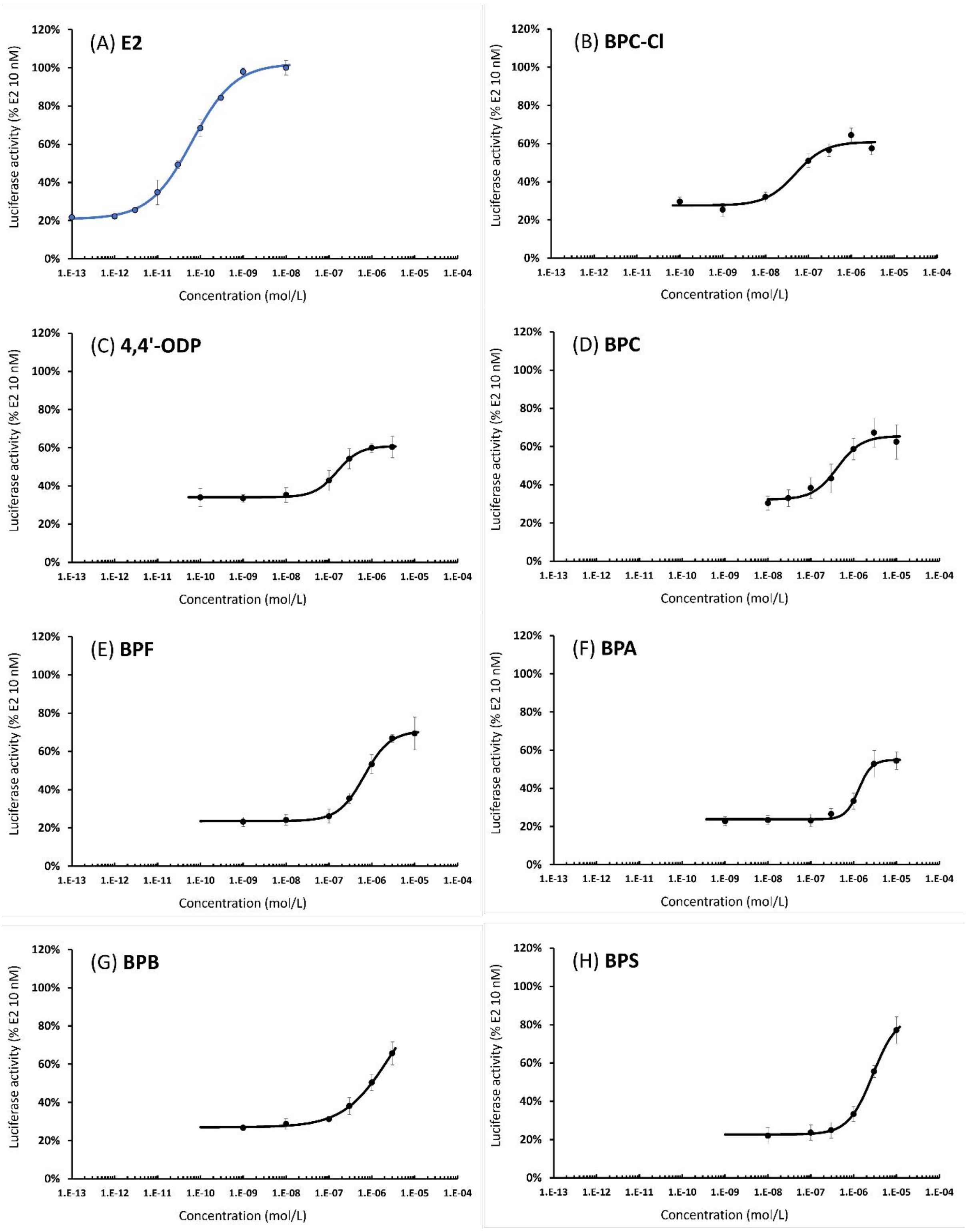

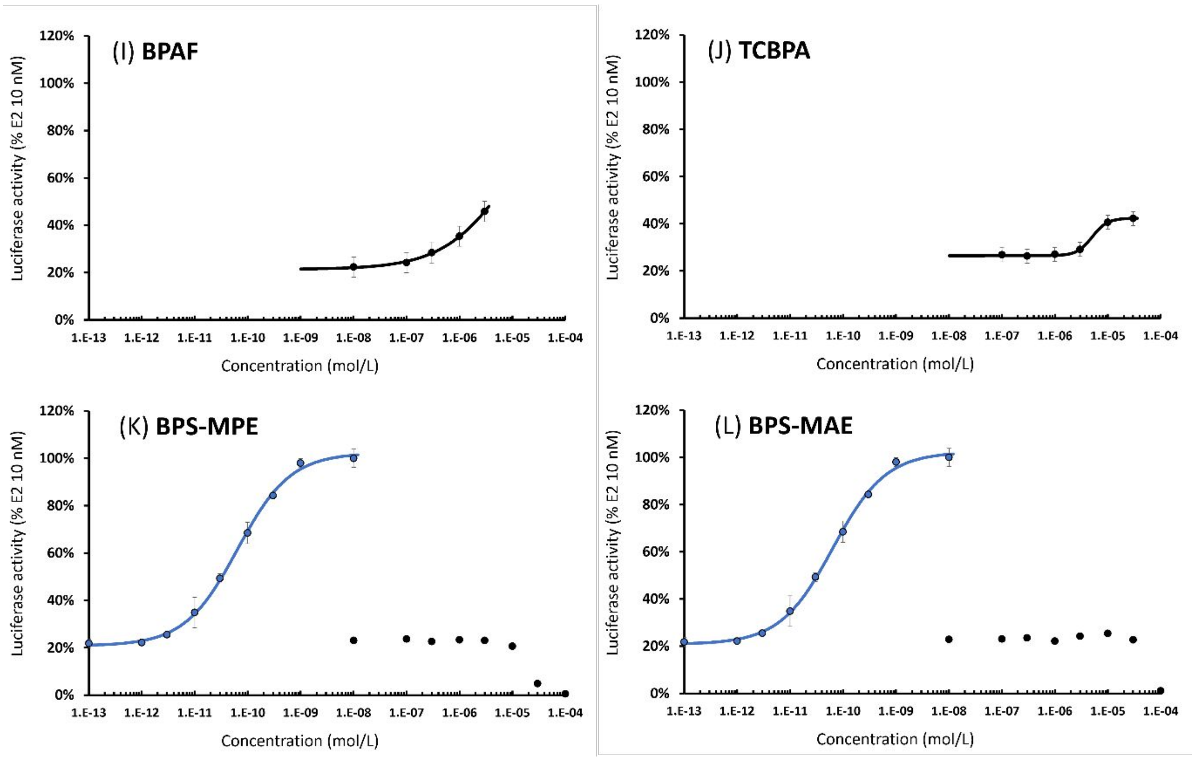
(I) BPAF
(J) TCBPA
(K) BPS-MPE
(L) BPS-MAE
Luciferase activity (% E2 10 nM)
Concentration (mol/L)
0%
20%
40%
60%
80%
100%
120%
1.E-13
1.E-12
1.E-11
1.E-10
1.E-09
1.E-08
1.E-07
1.E-06
1.E-05
1.E-04

REFERENCES

Arrokhman S, Luo Y-H, Lin P. 2023. Additive cardiotoxicity of a bisphenol mixture in zebrafish embryos: The involvement of calcium channel and pump. Ecotoxicol Environ Saf 263:115225; doi:10.1016/j.ecoenv.2023.115225.

Björnsdotter MK, Jonker W, Legradi J, Kool J, Ballesteros-Gómez A. 2017. Bisphenol A alternatives in thermal paper from the Netherlands, Spain, Sweden and Norway. Screening and potential toxicity. Science of The Total Environment 601–602:210–221; doi:10.1016/j.scitotenv.2017.05.171.

Blanc M, Rüegg J, Scherbak N, Keiter SH. 2019. Environmental chemicals differentially affect epigenetic-related mechanisms in the zebrafish liver (ZF-L) cell line and in zebrafish embryos. Aquatic Toxicology 215:105272; doi:10.1016/j.aquatox.2019.105272.

Brannen KC, Panzica-Kelly JM, Danberry TL, Augustine-Rauch KA. 2010. Development of a zebrafish embryo teratogenicity assay and quantitative prediction model. Birth Defects Research Part B: Developmental and Reproductive Toxicology 89:66–77; doi:10.1002/bdrb.20223.

Brown AR, Green JM, Moreman J, Gunnarsson LM, Mourabit S, Ball J, et al. 2019. Cardiovascular Effects and Molecular Mechanisms of Bisphenol A and Its Metabolite MBP in Zebrafish. Environ Sci Technol 53:463–474; doi:10.1021/acs.est.8b04281.

Catron TR, Keely SP, Brinkman NE, Zurlinden TJ, Wood CE, Wright JR, et al. 2019. Host Developmental Toxicity of BPA and BPA Alternatives Is Inversely Related to Microbiota Disruption in Zebrafish. Toxicological Sciences 167:468–483; doi:10.1093/toxsci/kfy261.

Chen M-Y, Ike M, Fujita M. 2002. Acute toxicity, mutagenicity, and estrogenicity of bisphenol-A and other bisphenols. Environmental Toxicology 17:80–86; doi:10.1002/tox.10035.

Chen P, Wang Q, Chen M, Yang J, Wang R, Zhong W, et al. 2018. Antagonistic Estrogenic Effects Displayed by Bisphenol AF and Perfluorooctanoic Acid on Zebrafish (Danio rerio) at an Early Developmental Stage. Environ Sci Technol Lett 5:655–661; doi:10.1021/acs.estlett.8b00559.

Chow WS, Chan WK, Chan KM. 2013. Toxicity assessment and vitellogenin expression in zebrafish (*Danio rerio*) embryos and larvae acutely exposed to bisphenol A, endosulfan, heptachlor, methoxychlor and tetrabromobisphenol A. J of Applied Toxicology 33:670–678; doi:10.1002/jat.2723.

Corrales J, Kristofco LA, Steele WB, Saari GN, Kostal J, Williams ES, et al. 2017. Toward the Design of Less Hazardous Chemicals: Exploring Comparative Oxidative Stress in Two Common Animal Models. Chem Res Toxicol 30:893–904; doi:10.1021/acs.chemrestox.6b00246.

Duan Z, Zhu L, Zhu L, Kun Y, Zhu X. 2008. Individual and joint toxic effects of pentachlorophenol and bisphenol A on the development of zebrafish (*Danio rerio*)

embryo. Ecotoxicology and Environmental Safety 71:774–780; doi:10.1016/j.ecoenv.2008.01.021.

Green JM, Metz J, Lee O, Trznadel M, Takesono A, Brown AR, et al. 2016. High-Content and Semi-Automated Quantification of Responses to Estrogenic Chemicals Using a Novel Translucent Transgenic Zebrafish. Environ Sci Technol 50:6536–6545; doi:10.1021/acs.est.6b01243.

Gu J, Wu J, Xu S, Zhang L, Fan D, Shi L, et al. 2020. Bisphenol F exposure impairs neurodevelopment in zebrafish larvae (*Danio rerio*). Ecotoxicology and Environmental Safety 188:109870; doi:10.1016/j.ecoenv.2019.109870.

Le Fol V, Aït-Aïssa S, Sonavane M, Porcher J-M, Balaguer P, Cravedi J-P, et al. 2017. *In vitro* and *in vivo* estrogenic activity of BPA, BPF and BPS in zebrafish-specific assays. Ecotoxicology and Environmental Safety 142:150–156; doi:10.1016/j.ecoenv.2017.04.009.

Makarova K, Siudem P, Zawada K, Kurkowiak J. 2016. Screening of Toxic Effects of Bisphenol A and Products of Its Degradation: Zebrafish (Danio rerio) Embryo Test and Molecular Docking. Zebrafish 13:466–474; doi:10.1089/zeb.2016.1261.

Martínez R, Herrero-Nogareda L, Van Antro M, Campos MP, Casado M, Barata C, et al. 2019. Morphometric signatures of exposure to endocrine disrupting chemicals in zebrafish eleutheroembryos. Aquatic Toxicology 214:105232; doi:10.1016/j.aquatox.2019.105232.

McCormick JM, Es TV, Cooper KR, White LA, Häggblom MM. 2011. Microbially Mediated O-Methylation of Bisphenol a Results in Metabolites with Increased Toxicity to the Developing Zebrafish (Danio rerio) Embryo. Environ Sci Technol 45:6567–6574; doi:10.1021/es200588w.

McCormick JM, Paiva MS, Häggblom MM, Cooper KR, White LA. 2010. Embryonic exposure to tetrabromobisphenol A and its metabolites, bisphenol A and tetrabromobisphenol A dimethyl ether disrupts normal zebrafish (*Danio rerio*) development and matrix metalloproteinase expression. Aquatic Toxicology 100:255–262; doi:10.1016/j.aquatox.2010.07.019.

Moreman J, Lee O, Trznadel M, David A, Kudoh T, Tyler CR. 2017. Acute Toxicity, Teratogenic, and Estrogenic Effects of Bisphenol A and Its Alternative Replacements Bisphenol S, Bisphenol F, and Bisphenol AF in Zebrafish Embryo-Larvae. Environ Sci Technol 51:12796–12805; doi:10.1021/acs.est.7b03283.

Mu X, Huang Y, Li X, Lei Y, Teng M, Li X, et al. 2018. Developmental Effects and Estrogenicity of Bisphenol A Alternatives in a Zebrafish Embryo Model. Environ Sci Technol 52:3222–3231; doi:10.1021/acs.est.7b06255.

Pelch K, Wignall JA, Goldstone AE, Ross PK, Blain RB, Shapiro AJ, et al. 2019. A scoping review of the health and toxicological activity of bisphenol A (BPA) structural analogues and functional alternatives. Toxicology 424:152235; doi:10.1016/j.tox.2019.06.006.

Qin J-Y, Jia W, Ru S, Xiong J-Q, Wang J, Wang W, et al. 2023. Bisphenols induce cardiotoxicity in zebrafish embryos: Role of the thyroid hormone receptor pathway. Aquat Toxicol 254:106354; doi:10.1016/j.aquatox.2022.106354.

Qiu W, Liu S, Chen H, Luo S, Xiong Y, Wang X, et al. 2021. The comparative toxicities of BPA, BPB, BPS, BPF, and BPAF on the reproductive neuroendocrine system of zebrafish embryos and its mechanisms. Journal of Hazardous Materials 406:124303; doi:10.1016/j.jhazmat.2020.124303.

Qiu W, Shao H, Lei P, Zheng C, Qiu C, Yang M, et al. 2018. Immunotoxicity of bisphenol S and F are similar to that of bisphenol A during zebrafish early development. Chemosphere 194:1–8; doi:10.1016/j.chemosphere.2017.11.125.

Qiu W, Zhao Y, Yang M, Farajzadeh M, Pan C, Wayne NL. 2016. Actions of Bisphenol A and Bisphenol S on the Reproductive Neuroendocrine System During Early Development in Zebrafish. Endocrinology 157:636–647; doi:10.1210/en.2015-1785.

Ren W-J, Wang Z, Yang X, Liu J, Yang Y, Chen Y, et al. 2017. Acute Toxicity Effect of Bisphenol A and Its Analogues on Adult and Embryo of Zebrafish. stynchjxb 33:372–378; doi:10.11934/j.issn.1673-4831.2017.04.011.

Riu A, McCollum CW, Pinto CL, Grimaldi M, Hillenweck A, Perdu E, et al. 2014. Halogenated Bisphenol-A Analogs Act as Obesogens in Zebrafish Larvae (Danio rerio). Toxicol Sci 139:48–58; doi:10.1093/toxsci/kfu036.

Saili KS, Corvi MM, Weber DN, Patel AU, Das SR, Przybyla J, et al. 2012. Neurodevelopmental low-dose bisphenol A exposure leads to early life-stage hyperactivity and learning deficits in adult zebrafish. Toxicology 291:83–92; doi:10.1016/j.tox.2011.11.001.

Song M, Liang D, Liang Y, Chen M, Wang F, Wang H, et al. 2014. Assessing developmental toxicity and estrogenic activity of halogenated bisphenol A on zebrafish (*Danio rerio*). Chemosphere 112:275–281; doi:10.1016/j.chemosphere.2014.04.084.

Tišler T, Krel A, Gerželj U, Erjavec B, Dolenc MS, Pintar A. 2016. Hazard identification and risk characterization of bisphenols A, F and AF to aquatic organisms. Environmental Pollution 212:472–479; doi:10.1016/j.envpol.2016.02.045.

Üstündağ ÜV, Ünal İ, Ateş PS, Alturfan AA, Yiğitbaşı T, Emekli-Alturfan E. 2017. Bisphenol A and di(2-ethylhexyl) phthalate exert divergent effects on apoptosis and the Wnt/β-catenin pathway in zebrafish embryos: A possible mechanism of endocrine disrupting chemical action. Toxicol Ind Health 33:901–910; doi:10.1177/0748233717733598.

Wang X, Dong Q, Chen Y, Jiang H, Xiao Q, Wang Y, et al. 2013. Bisphenol A affects axonal growth, musculature and motor behavior in developing zebrafish. Aquatic Toxicology 142–143:104–113; doi:10.1016/j.aquatox.2013.07.011.

Wu M, Xu H, Shen Y, Qiu W, Yang M. 2011. Oxidative stress in zebrafish embryos induced by short-term exposure to bisphenol A, nonylphenol, and their mixture. Environmental Toxicology and Chemistry 30:2335–2341; doi:10.1002/etc.634.

Yang Q, Yang X, Liu J, Ren W, Chen Y, Shen S. 2017. Effects of BPF on steroid hormone homeostasis and gene expression in the hypothalamic–pituitary–gonadal axis of zebrafish. Environ Sci Pollut Res 24:21311–21322; doi:10.1007/s11356-017-9773-z.

Zhang D, Zhou E, Yang Z. 2017. Waterborne exposure to BPS causes thyroid endocrine disruption in zebrafish larvae. PLOS ONE 12:e0176927; doi:10.1371/journal.pone.0176927.
